# mTOR activates the VPS34–UVRAG complex to regulate autolysosomal tubulation and cell survival

**DOI:** 10.15252/embj.201590992

**Published:** 2015-07-02

**Authors:** Michael J Munson, George FG Allen, Rachel Toth, David G Campbell, John M Lucocq, Ian G Ganley

**Affiliations:** 1 MRC Protein Phosphorylation and Ubiquitylation Unit, College of Life Sciences, University of DundeeDundee, UK; 2 School of Medicine, University of St AndrewsSt Andrews, UK

**Keywords:** lysosome, mTOR, tubule, UVRAG, VPS34

## Abstract

Lysosomes are essential organelles that function to degrade and recycle unwanted, damaged and toxic biological components. Lysosomes also act as signalling platforms in activating the nutrient-sensing kinase mTOR. mTOR regulates cellular growth, but it also helps to maintain lysosome identity by initiating lysosomal tubulation through a process termed autophagosome-lysosome reformation (ALR). Here we identify a lysosomal pool of phosphatidylinositol 3-phosphate that, when depleted by specific inhibition of the class III phosphoinositide 3-kinase VPS34, results in prolonged lysosomal tubulation. This tubulation requires mTOR activity, and we identified two direct mTOR phosphorylation sites on UVRAG (S550 and S571) that activate VPS34. Loss of these phosphorylation sites reduced VPS34 lipid kinase activity and resulted in an increase in number and length of lysosomal tubules. In cells in which phosphorylation at these UVRAG sites is disrupted, the result of impaired lysosomal tubulation alongside ALR activation is massive cell death. Our data imply that ALR is critical for cell survival under nutrient stress and that VPS34 is an essential regulatory element in this process.

See also: **Y Chen & L Yu** (September 2015)

## Introduction

Intracellular membrane compartmentalisation, to form organelles, is vital to enable distinct biological processes to occur in a controlled manner. Cellular components, such as lipids and proteins, must be trafficked to and from these organelles in order for these processes to occur. A fundamental question in cell biology concerns how specific compartment identity is retained in spite of perpetual transport to and from each organelle. A prime example of this is the lysosome, a digestive organelle that is often considered the end point of many intracellular transport pathways including endocytosis, phagocytosis and autophagy (Appelqvist *et al*, 2013; Puertollano, 2014). In spite of all this incoming material, lysosomes still maintain their identity and basic structure. One mechanism by which lysosomes achieve this is through a process termed autophagosome-lysosome reformation (ALR), which was identified from studies following long-term starvation and the nutrient-sensing kinase mTOR (Yu *et al*, 2010). Starvation through loss of amino acids leads to inactivation of mTOR and induction of autophagy (Noda & Ohsumi, 1998). This in turn induces bulk transport of autophagosomes and their contents to lysosomes, generating large autolysosomes. Degradation of their contents replenishes amino acids to allow synthesis of essential proteins to aid in survival. At a critical point, the production of amino acids from degraded protein is sufficient to re-activate mTOR, an important regulator of ALR that drives reformation of lysosomes from autolysosomes via membrane tubulation (Chen & Yu, 2013).

Multiple pathways that traffic material to the lysosomes require the lipid kinase VPS34, the only known class III phosphoinositide 3-kinase (Backer, 2008; Raiborg *et al*, 2013). VPS34 catalyses the phosphorylation of phosphatidylinositol (PI) at the three position of the inositol ring to produce phosphatidylinositol 3-phosphate (PI(3)P). PI(3)P then acts as a signalling molecule to recruit downstream proteins, such as those involved in membrane tethering and fusion, mainly through PI(3)P-specific binding domains such as the PX or FYVE domains (Di Paolo & De Camilli, 2006; Lemmon, 2008). VPS34 regulation of multiple pathways is enabled by binding to distinct protein complexes, which allow further spatiotemporal regulation of the different trafficking pathways. In mammalian cells, there are at least two distinct VPS34 complexes that each contain VPS34, VPS15 and BECLIN1 and either ATG14L/BARKOR or UVRAG (Kihara *et al*, 2001; Itakura *et al*, 2008; Sun *et al*, 2008). The ATG14L-containing complex is primarily thought to function in the autophagic pathway, while the UVRAG-containing complex functions in the endocytic pathway (Simonsen & Tooze, 2009; Funderburk *et al*, 2010).

By nature of its involvement in lysosomal trafficking pathways, VPS34 has an indirect role on lysosomal function; however, we were curious to examine whether VPS34 and its product PI(3)P play a more direct role in regulating lysosomal function, thus providing a mechanism for coordination of not only these distinct trafficking pathways, but also their common end point.

Here, we uncover a role for VPS34 and PI(3)P at the lysosome and identify mTOR phosphorylation sites on the VPS34 binding partner UVRAG. Loss of these sites reduces VPS34 activity, leading to persistent lysosomal tubulation and a failure to regenerate normal lysosomes that results in increased cell death following starvation.

## Results

**Figure 1:**
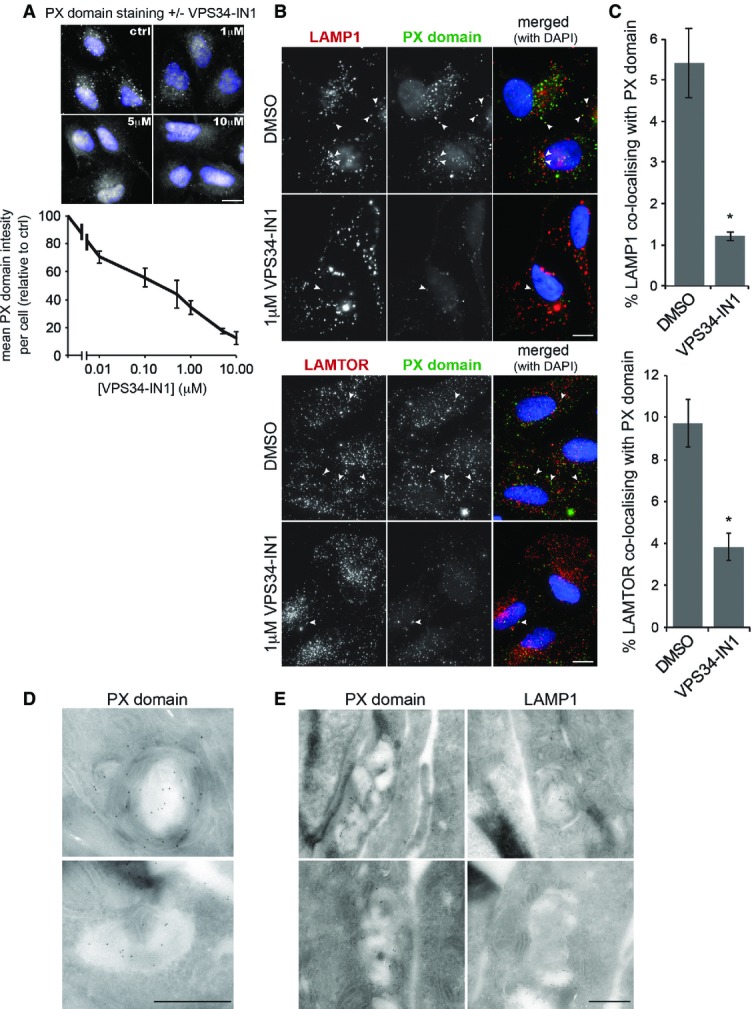
PI(3)P is present at the lysosome U2OS cells were treated with indicated concentrations of VPS34-IN1 for 1 h, fixed and stained with a recombinant PX domain Alexa Fluor-594 conjugate. Graph represents mean PX domain staining following titration of VPS34-IN1 in U2OS cells for 1 h ± SEM for *n* = 3 independent experiments. Scale bar, 10 μm.

To determine the role of VPS34 in lysosomal function, we titrated the highly specific VPS34 inhibitor, VPS34-IN1 (Bago *et al*, 2014), to generate a range of PI(3)P levels within the cell (Fig1A). In order to visualise intracellular PI(3)P, we stained fixed and permeabilised cells with fluorescently tagged recombinant PX domain derived from p40phox (Ellson *et al*, 2001). This protein domain is specific for PI(3)P, and we prefer this method of staining as it avoids artefacts and dominant-negative effects that can occur by overexpressing high-affinity PI(3)P-binding domains in live cells. Using this method, we found that 5 μM VPS34-IN1 reduced the PX domain staining by 90%, while 1 μM reduced it by 60%. This is in good agreement with our previous study showing that the inhibitor blocked membrane association of intracellularly expressed tandem FYVE domains (Bago *et al*, 2014). PI(3)P is known to function in endocytic pathway sorting and in autophagy initiation, both of which indirectly impinge upon lysosomal function. However, there is little evidence to suggest that VPS34 and its product PI(3)P also act directly at the lysosome itself. To examine whether PI(3)P is present on lysosomes, we co-stained cells with the recombinant PX domain and lysosomal associated membrane protein 1 (LAMP1) and lysosomal adaptor and mTOR regulator (LAMTOR), both endogenous lysosomal markers (Fig1B). We identified a small but significant pool of PX domain staining localised to both LAMP1 and LAMTOR structures in cells (Fig1C), indicating that PI(3)P is indeed present on lysosomes. We further analysed another marker, CD63 (also known as LAMP3), which is enriched on lysosomes and again found significant co-localisation with the PX domain (Supplementary Fig S1). Consistent with previous publications, we observed a large proportion of the PX domain to label early endosomes (Ellson *et al*, 2001; Supplementary Fig S1). We are currently unable to tell whether it is a subset of lysosomes that contain PI(3)P or, more likely given the evidence presented later, whether it is more a temporal localisation with lysosomes acquiring PI(3)P at certain stages in their lifetime. We found that treatment of cells with 1 μM VPS34-IN1 dramatically reduced PX-LAMP1 colocalisation by 80%, which, given the 60% reduction of total PX domain staining (Fig1A), suggests that this lysosomal pool of PI(3)P is particularly sensitive to VPS34 inhibition. Additional evidence for a lysosomal pool of PI(3)P was provided by transition electron microscopy. We labelled cell sections with recombinant GST-tagged PX domain followed by secondary anti-GST conjugated to gold particles and found a small number of labelled structures that resemble lysosomes in that they were multilamellar and often contained pleiotropic membranous structures in their lumen (Fig1D). We were unable to perform double-labelling experiments, but found that LAMP1 also labelled similar membranous profiles, further supporting the idea that they are lysosomal (Fig1E). Taken together, the microscopy data strongly suggest PI(3)P is present on lysosomes.

U2OS cells were treated for 1 h as indicated and stained for LAMP1 or LAMTOR and PI(3)P utilising the PX domain conjugate; arrowheads indicate co-localising punctate structures. Scale bar, 10 μm.

Quantitation of LAMP1 or LAMTOR and PX domain co-localisation from (B), mean ± SEM for *n* = 3 independent experiments. Significance was determined by Student’s *t*-test, *P < 0.05.

Transition electron micrograph sections of U2OS cells grown in complete medium and labelled with recombinant GST-PX domain and anti-GST gold conjugate. Scale bar, 500 nm.

Electron micrograph sections of U2OS cells as in (D) labelled for PX domain or LAMP1. Scale bar, 500 nm.

**Figure 2:**
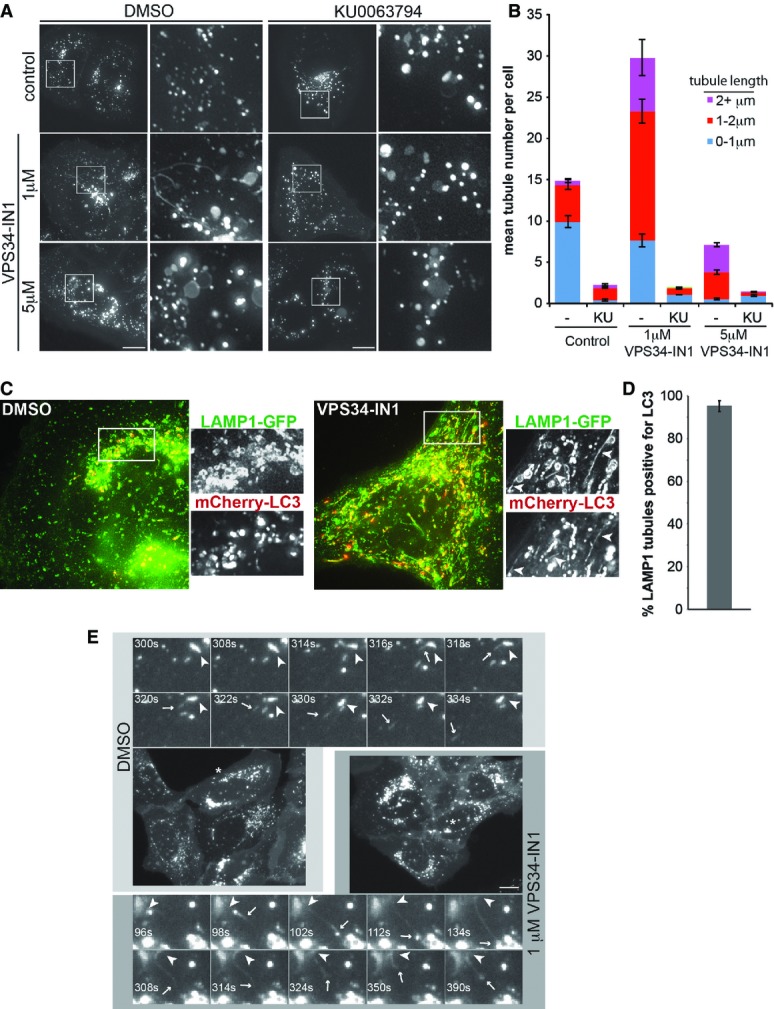
Inhibition of VPS34 alters autolysosomal morphology Images from live U2OS cells stably expressing LAMP1-mCherry that were treated as indicated for 1 h. White squares denote magnified regions shown to the right. Scale bar, 10 μm.

Given the dynamic nature of lysosomes and to preserve tubular elements that can be disrupted by fixation (Sridhar *et al*, 2013), we analysed lysosomal structures in live U2OS cells stably expressing low levels of LAMP1-mCherry (Fig2). Inhibition of VPS34 had a clear effect on lysosomal morphology, both at 1 and at 5 μM VPS34-IN1 (Fig2A) with lysosomes appearing larger in general. Larger lysosomes are seen upon PIKfyve inhibition, the lipid kinase that uses PI(3)P to generate PI(3,5)P_2_, a phospholipid involved in regulating endosomal and lysosomal size (Jefferies *et al*, 2008). It is therefore possible that this larger lysosomal size is due to reduced PI(3,5)P_2_ levels upon VPS34-IN1 treatment. Remarkably at 1 μM VPS34-IN1, which inhibits lysosomal PI(3)P levels, a large increase in lysosomal tubulation occurred (Fig2A, right panels, and B). These tubules are reminiscent of ALR where lysosomes regenerate following starvation and reactivation of mTOR signalling (Yu *et al*, 2010). To determine whether the VPS34-IN1-induced tubules occur through a related process, cells were treated in combination with the specific mTOR inhibitor KU0063794 (shortened to KU here; García-Martínez *et al*, 2009; Fig2A, left panels, and B). mTOR inhibition is known to increase lysosomal size, and this had no effect on the large lysosomes seen upon VPS34-IN1 treatment. However, mTOR inhibition completely abolished the tubules formed upon 1 μM VPS34-IN1 treatment, implying these tubules are linked to ALR. We also used GFP-LAMTOR as an additional lysosomal marker and found this behaved in a very similar fashion to LAMP1-mCherry with respect to tubule formation (Supplementary Fig S2).

Quantitation from (A) of mean tubules per cell and tubule length as indicated ± SEM for *n* = 3 independent experiments.

Live cell images of U2OS cells expressing LAMP1-GFP and mCherry-LC3 grown in complete medium and treated with DMSO or 1 μM VPS34-IN1 for 1 h. Arrow heads in boxed regions highlight tubular co-localisation of LAMP1-GFP and mCherry-LC3.

Quantitation of co-localisation of LAMP1 tubules with LC3 from (C). Mean ± SEM for *n *=* *3 independent experiments.

Still images from live cell microscopy shown in Supplementary Movie S1A and B. For control (DMSO), magnified frames are shown above the cell from the area marked with an asterisk. For VPS34-IN1 treatment, frames are shown below. Arrowheads indicate initiation site of LAMP1 tubules, while arrows indicate the leading edge of tubules. Scale bar, 10 μm.

As ALR initiates from autolysosomes, we further analysed the nature of the LAMP1 tubules by stably expressing mCherry-LC3 in our LAMP1-GFP cells (Fig2C). Given its relative lysosomal stability, mCherry derived from mCherry-LC3 will accumulate over time in autolysosomes due to basal autophagy (Klionsky *et al*, 2012). We therefore analysed tubule formation in these cells upon VPS34-IN1 treatment and found that over 90% of the LAMP1-GFP tubules also contained the mCherry signal (Fig2C and D). This demonstrates that the tubules are forming from autolysosomes.

The dynamics of lysosome tubulation is shown in more detail in Fig2E, and the reader is encouraged to observe the movies that these images are derived from (Supplementary Movie S1A and B). Under control conditions, a small tubule can be seen to form on the indicated LAMP1 structure (arrowhead, upper panels in Fig2E), and following approximately 30 s, the tubule buds off and becomes an independent structure (arrow, upper panels in Fig2E). In contrast, treatment with VPS34-IN1 results in the formation of a tubule that grows and extends towards another LAMP1 compartment (arrow, lower panels in Fig2E). The tubule persists and “springs” back after around 300 s, implying that scission of the tubule is disrupted.

Given that VPS34 inhibition with VPS34-IN1 prolonged auto-lysosomal tubulation, which in turn was blocked by mTOR inhibition, we explored the possibility that mTOR may directly control VPS34 activity. To analyse this further, we looked at the VPS34 lipid kinase complex in more detail.

### mTOR phosphorylates the UVRAG–VPS34 lipid kinase complex

**Figure 3:**
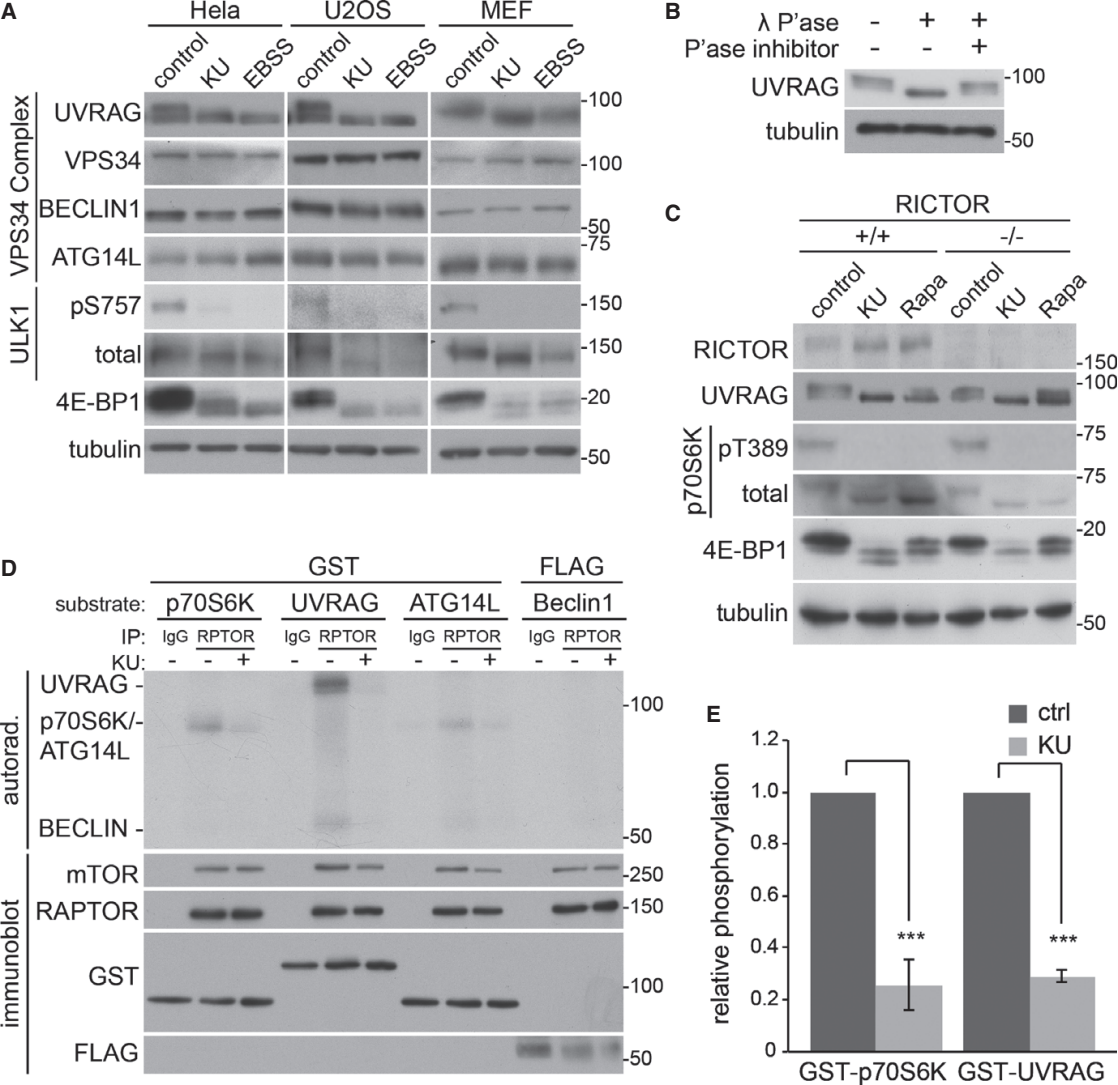
mTORC1 phosphorylates the UVRAG–VPS34 lipid kinase complex HeLa, U2OS and MEFs were treated in complete medium (control), 1 μM KU0063794 (KU) or EBSS for 1 h (MEFs) or 2 h (HeLa, U2OS) prior to lysis and immunoblot.

To investigate the possibility that mTOR regulates VPS34, we examined lysates from three different cell lines (HeLa, U2OS and MEF) grown under control conditions, treated with the mTOR inhibitor KU or incubated in amino acid-free Earle’s balanced salt solution (EBSS), which also results in mTOR inhibition (Fig3A). Immunoblot analysis confirmed mTOR inhibition by KU and EBSS, as indicated by a loss of ULK1 phosphorylation at the mTOR site (serine 757) and increased electrophoretic mobility (due to loss of phosphorylation) of another known mTOR substrate, 4E-BP1. As VPS34 exists in two core complexes containing either UVRAG or ATG14L, we analysed these along with VPS34 and BECLIN1. No notable change was observed in any VPS34 component except for the protein UVRAG, which ran as a doublet under normal conditions, but exhibited loss of the upper band upon mTOR inhibition. As an electrophoretic mobility shift can be indicative of phosphorylation, we confirmed this by treating MEF lysates grown under normal conditions with λ phosphatase that has broad non-specific phosphatase activity (Zhuo *et al*, 1993) (Fig3B). λ Phosphatase caused a mobility shift but this was prevented in the presence of phosphatase inhibitors, confirming that UVRAG is indeed phosphorylated under conditions where mTOR is active.

Cell lysate from MEFs grown in complete medium was treated in the presence or absence of lambda phosphatase with or without phosphatase inhibitors for 30 min at 30°C.

RICTOR +/+ and −/− MEFs were grown in complete medium (control), 1 μM KU or EBSS for 1 h prior to lysis.

Endogenous RAPTOR (RPTOR) or IgG control was immunoprecipitated from HEK293 cells and utilised for an *in vitro* kinase assay (Materials and Methods) with substrates GST-p70S6K D236A, GST-UVRAG, GST-ATG14L or FLAG-BECLIN1 in the presence or absence of KU.

Quantitation of relative phosphorylation of GST-p70S6K and GST-UVRAG in the presence or absence of KU from (D). Data represents mean ± SEM for *n* = 3 independent experiments. Significance was determined by Student’s *t*-test, ***P < 0.001. Source data are available online for this figure.

mTOR is present in two distinct complexes, mTORC1 and mTORC2, both of which can be inhibited by the KU compound (García-Martínez *et al*, 2009). To determine mTORC2 involvement, we examined UVRAG mobility in SDS–PAGE gels with lysates from mTORC2-deficient, RICTOR-null MEFs. UVRAG demonstrated a comparable mobility shift in both RICTOR-positive and RICTOR-negative cells, indicating mTORC2 is not required (Fig3C). In contrast to KU, the specific mTORC1 inhibitor rapamycin only partially blocked the mobility shift of UVRAG, as it did for 4E-BP1 also, indicating that residual UVRAG phosphorylation may be due to the rapamycin-insensitive properties of mTORC1 (Choo *et al*, 2008; Kang *et al*, 2013).

To confirm that mTOR can phosphorylate UVRAG, we carried out *in vitro* kinase assays using RAPTOR immunoprecipitation (IP) as a source of endogenous mTORC1 complex. For substrates, we used recombinant GST-UVRAG as well as the VPS34 binders GST-ATG14L and FLAG-BECLIN1. We also included GST-p70S6K D236A (kinase dead), a known mTORC1 substrate as a positive control (Fig3D). Autoradiography to detect ^32^P incorporation into substrates revealed that UVRAG is highly phosphorylated by mTORC1 at a level that was greater than the known substrate p70S6K. We observed no phosphorylation by mTORC1 on BECLIN1 and a small degree of ATG14L phosphorylation, which is in support of the recent observation that mTOR can regulate ATG14L (Yuan *et al*, 2013). Interestingly, we did observe a phosphorylated band at the position of BECLIN1 in the UVRAG samples, which could imply that mTOR can phosphorylate BECLIN1 when it is part of the UVRAG complex. Further work will be needed to clarify this. Phosphorylation of each substrate was sensitive to KU treatment, indicating that mTOR is the major phosphorylating kinase. Indeed, UVRAG sensitivity to KU was equivalent to p70S6K, further implicating UVRAG as a direct mTOR substrate (Fig3E). Given the mTOR-dependent electrophoretic mobility shift of UVRAG derived from cells and *in vitro* phosphorylation by mTOR, we examined this event in further detail.

### mTOR phosphorylates UVRAG at serine 550 and serine 571

**Figure 4:**
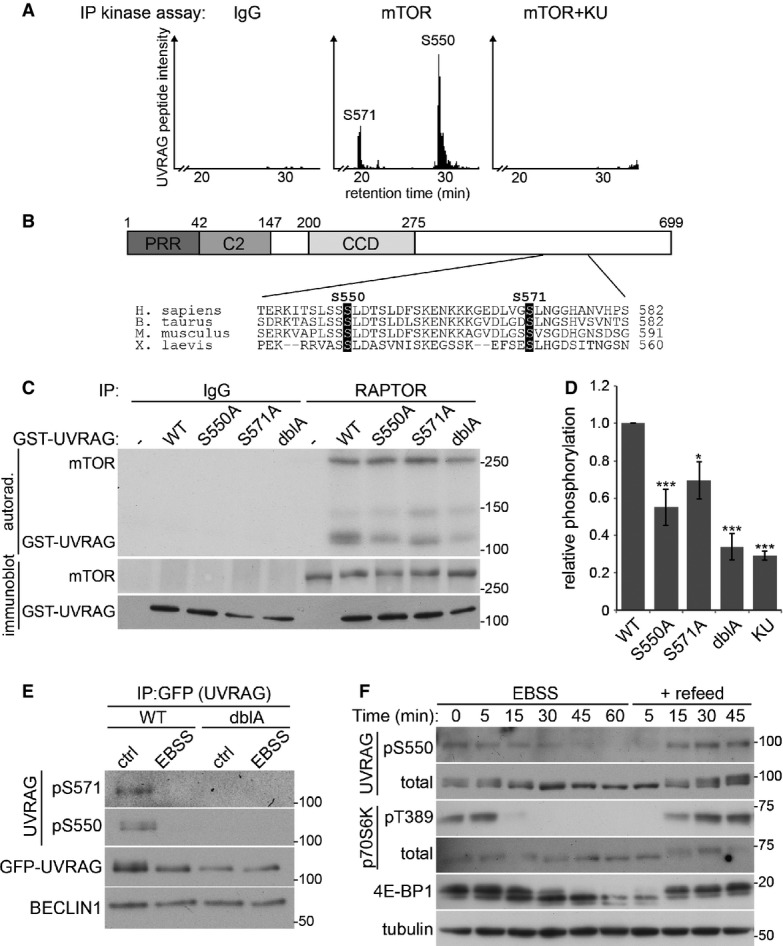
mTOR phosphorylates UVRAG at serine 550 and serine 571 Extracted ion chromatogram of peptide K543–K559 (S550) and K564–R595 (S571) following *in vitro* kinase assay of GST-UVRAG with IgG control or endogenous mTOR in the presence or absence of 1 μM KU0063794 (KU).

To identify the phosphorylation sites on UVRAG, two approaches were undertaken. GST-UVRAG was incubated *in vitro* with endogenous mTOR and phosphorylated in a non-radioactive kinase assay in the absence or presence of KU. Alternatively, MEFs stably expressing FLAG-UVRAG were treated with/without KU and UVRAG immunoprecipitated using FLAG agarose. In each experiment, samples were separated by SDS–PAGE and the Coomassie-stained UVRAG band was excised for phospho-peptide analysis by mass spectrometry. The *in vitro* kinase assay identified two phosphorylated sites that were abolished upon KU treatment, serine 550 (S550) and serine 571 (S571, Fig4A). Analysis of UVRAG immunoprecipitated from cells also identified S550 and S571 as being phosphorylated in cells and sensitive to KU treatment. From the immunoprecipitated UVRAG only, we also identified two additional sites that were phosphorylated at serine 498 and threonine 518. However, unlike S550 and S571, these sites were insensitive to KU treatment and a UVRAG mutant with these residues substituted to alanine was still phosphorylated *in vitro* by mTOR, implying that another kinase mediates phosphorylation at these sites (Supplementary Fig S3). Combined, the *in vitro* and cell data clearly indicate that mTORC1 phosphorylates UVRAG at two distinct sites. The domain structure of UVRAG is represented in Fig4B with an alignment of human UVRAG residues 540–582, the region where phosphorylated serines were identified. Both S550 and S571 residues are positioned with a +1 leucine that is favourable for mTOR-mediated phosphorylation (Hsu *et al*, 2011) and are well conserved across higher eukaryotes, further supporting that these are mTOR sites.

Domain structure of UVRAG containing an N-terminal proline-rich region (PRR), C2 domain and a coiled-coil domain (CCD). Sequence alignment of human UVRAG 540–582 indicates S550 and S571 (highlighted) which are conserved between species within the C-terminal domain.

*In vitro* kinase assay of endogenous mTORC1 by RAPTOR immunoprecipitation from HEK293 cells with GST-UVRAG wild-type (WT), S550A, S571A or S550A+S571A double (dblA) mutants.

Quantitation of (C), bars represent mean ^32^P incorporation ± SEM of *n *=* *4 independent experiments. Statistical analysis was carried out by one-way ANOVA and Dunnett’s multiple comparison test to the WT control, *P < 0.05 and ***P < 0.001.

U2OS cells stably expressing WT or dblA GFP-UVRAG were incubated in complete medium (ctrl) or EBSS for 2 h prior to immunoprecipitation with anti-GFP sepharose and immunoblotting with phospho-specific antibodies.

Wild-type MEFS were washed twice and incubated in EBSS for 1 h prior to refeed with complete medium for 45 min and lysed at time points indicated. Source data are available online for this figure.

To provide more evidence of S550 and S571 phosphorylation, the serine residues were mutated to alanines that cannot be phosphorylated as they lack a hydroxyl group. The GST-UVRAG alanine mutants were incubated *in vitro* with endogenous mTORC1 that was immunoprecipitated via RAPTOR (Fig4C and D). Mutation of S550 to alanine (S550A) reduced phosphorylation *in vitro*. A reduction was also observed when S571 was mutated to alanine (S571A) and UVRAG with combined S550A and S571A mutations (dblA) resulted in an additive loss of ^32^P incorporation. UVRAG dblA reduced mTOR-mediated phosphorylation by ∼70%, similar to that of wild-type (WT) UVRAG phosphorylation in the presence of KU (Fig4D). This implies that S550 and S571 are the primary mTORC1-mediated phosphorylation sites. The residual KU-insensitive ^32^P incorporation into UVRAG likely results from a contaminating kinase associated with the mTOR IP.

To confirm that UVRAG is phosphorylated at S550 and S571 in cells, we generated phospho-specific antibodies to these sites. IP of stably expressed GFP-UVRAG from cells under control conditions produced immunoreactivity with both the phospho-specific S550 and the phospho-specific S571 antibodies. Importantly, this signal was lost when cells were incubated with EBSS to inhibit mTOR activity (Fig4E). As a control, cells expressing GFP-UVRAG dblA at a similar level to WT GFP-UVRAG failed to produce any reactivity with the phospho-specific antibodies under any condition. Importantly, WT GFP-UVRAG displayed the same electrophoretic mobility shift as endogenous UVRAG upon mTOR inhibition while the dblA mutant did not, indicating that the UVRAG band shift represents dephosphorylation at the S550 and S571 residues.

We were also able to detect S550 phosphorylation on endogenous UVRAG in cell lysates (Fig4F). Satisfyingly, the phospho-S550 kinetics closely matched the UVRAG band shift as well as that of the known mTOR substrates 4E-BP1 and p70S6K, showing that UVRAG behaves as a conventional mTOR substrate.

### UVRAG phosphorylation enhances VPS34 lipid kinase activity

**Figure 5:**
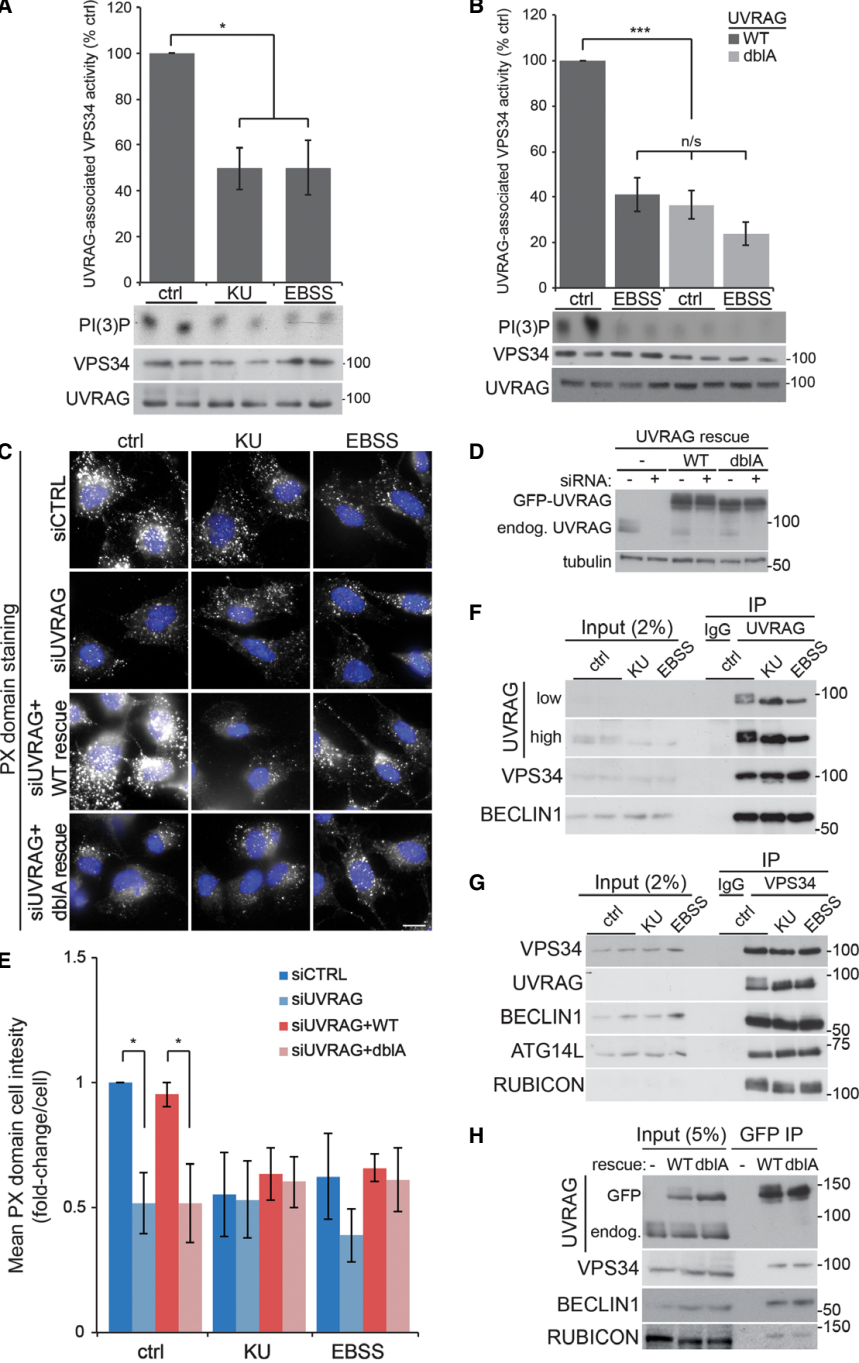
UVRAG phosphorylation enhances VPS34 lipid kinase activity A U2OS cells were incubated in complete medium (ctrl), 1 μM KU0063794 (KU) or EBSS for 2 h. Endogenous UVRAG was immunoprecipitated and associated VPS34 kinase activity determined by *in vitro* kinase assay (see Material and Methods). Graph represents mean VPS34 activity (following normalisation to VPS34 protein level) ± SEM for *n* = 3 independent experiments; significance was determined by one-way ANOVA and Dunnett’s multiple comparison test to the control, *P < 0.05.

UVRAG has been shown to enhance VPS34 activity (Liang *et al*, 2006; Sun *et al*, 2010). Given that our original observation showed that VPS34 activity was important in mTOR-dependent lysosomal tubulation (Fig2), we examined whether mTOR phosphorylation of UVRAG altered VPS34 activity. We first analysed endogenous UVRAG-associated VPS34 activity in an *in vitro* lipid kinase assay to measure PI(3)P production (Fig5A). Endogenous UVRAG was immunoprecipitated from cells under control conditions or those with mTOR inhibition and loss of UVRAG phosphorylation (either directly by KU treatment or indirectly through EBSS incubation). A significant drop in VPS34 activity of 50% was observed in U2OS cells upon mTOR inhibition, with KU treatment and EBSS incubation having a similar effect on activity. To see whether this reduction in VPS34 activity was due to loss of UVRAG–mTOR phosphorylation, we carried out similar experiments in cells stably expressing WT or dblA FLAG-UVRAG (Fig5B). Exogenous UVRAG was immunoprecipitated from cells, treated as in Fig5A, using the FLAG tag. WT FLAG-UVRAG behaved very similar to endogenous UVRAG in that the associated VPS34 activity was reduced by 60% upon mTOR inhibition with EBSS. The dblA mutant also displayed similar VPS34 activity upon EBSS treatment; however, critically, this activity did not increase under control conditions where mTOR is active. This suggests that S550 and S571 are essential to mediate the increased VPS34 activity under control conditions with complete medium.

B U2OS cells stably expressing wild-type (WT) or S550A+S571A (dblA) FLAG-UVRAG were incubated in complete medium (ctrl) or EBSS for 2 h prior to IP with FLAG agarose and *in vitro* kinase assay of associated VPS34 activity. Graph represents mean VPS34 activity ± SEM for *n* = 4 independent experiments; significance was determined by two-way ANOVA and Bonferroni post-tests, ***P < 0.001.

C Control MEFs or those stably expressing WT or dblA GFP-UVRAG were transfected with 100 nM UVRAG siRNA 40 h prior to treatment. Cells were treated as ctrl, KU or EBSS for 1 h prior to fixation and staining for PI(3)P with PX domain conjugate. Scale bar, 10 μm.

D Example blot of UVRAG siRNA knockdown and rescue as in (C).

E Quantitation of (C) where bars represent mean PX domain staining ± SEM for *n* = 4 independent experiments, significance was determined by two-way ANOVA and Bonferroni post-tests, *P < 0.05.

F, G IP of endogenous (F) UVRAG or (G) VPS34 from U2OS cells following ctrl, KU or EBSS treatment for 2 h.

H HeLa cells stably expressing WT or dblA GFP-UVRAG were incubated in complete medium prior to IP with GFP sepharose beads. Source data are available online for this figure.

To support the *in vitro* VPS34 activity data, we used the PX domain staining method to visualise PI(3)P in cells (Fig5C–E). To look at PI(3)P levels associated with UVRAG phosphorylation, we treated cells with KU and EBSS to inhibit mTOR. As with the *in vitro* VPS34 assays, inhibition of mTOR in cells resulted in a 50% loss of PX domain staining, indicating a reduction in cellular VPS34 activity (Fig5C, top panels, and E). To confirm that the reduction in PI(3)P staining was due to loss of UVRAG phosphorylation, we used siRNA to deplete endogenous UVRAG in cells stably expressing similar levels of either WT or dblA siRNA-resistant GFP-UVRAG (Fig5D). Loss of UVRAG alone caused a reduction in PX domain staining, supporting previous observations that UVRAG is important for VPS34 activity (Liang *et al*, 2006; Sun *et al*, 2010). This loss was similar to control-depleted cells treated with KU or EBSS and was not further reduced upon mTOR inhibition (Fig5C and E). Rescue of the UVRAG siRNA with expression of resistant WT UVRAG restored PX domain staining levels to that seen in control cells, ruling out UVRAG siRNA off-target effects. By contrast, expression of a similar level of dblA UVRAG failed to restore PX domain staining seen in control cells and displayed similar levels to the UVRAG-only depleted cells. Taken together with the *in vitro* data, this suggests that mTOR-mediated UVRAG phosphorylation at S550 and S571 enhances VPS34 activity. Consistent with the data presented here, mTOR inhibition has previously been shown to inhibit total levels of cellular PI(3)P (Byfield *et al*, 2005; Russell *et al*, 2013; Yuan *et al*, 2013); our data now demonstrate this inhibition is due to UVRAG dephosphorylation. It is important to note that mTOR inhibition does not reduce all pools of PI(3)P. The autophagy-initiating VPS34 complex, which contains ATG14L instead of UVRAG, is enhanced under these conditions, though the increase in autophagic PI(3)P is small compared to the total pool.

Given that the molecular make-up of the different VPS34 complexes is important for lipid kinase activity, it was important to check whether the VPS34-associated proteins changed upon UVRAG phosphorylation. We immunoprecipitated endogenous UVRAG from cells under control conditions or those of mTOR inhibition with KU or EBSS (Fig5F) and found that a similar amount of VPS34 and BECLIN1 co-immunoprecipitated regardless of the condition, suggesting phosphorylation does not alter UVRAG binding to the core VPS34 complex. Next, we immunoprecipitated endogenous VPS34 under similar conditions (Fig5G) and found that mTOR inhibition did not alter the amount of associating UVRAG, BECLIN1, ATG14L or RUBICON, suggesting UVRAG phosphorylation does not alter VPS34 complex stoichiometry. Finally, we immunoprecipitated stably expressed GFP-UVRAG from cells and found a similar amount of VPS34, BECLIN1 and RUBICON co-immunoprecipitating with either WT or the phosphorylation-deficient dblA mutant (Fig5H). Therefore, our data suggest the reduction in VPS34 activity upon loss of UVRAG dephosphorylation is not due to disruption of established VPS34 binding partners.

### UVRAG phosphorylation alters lysosomal morphology

**Figure 6:**
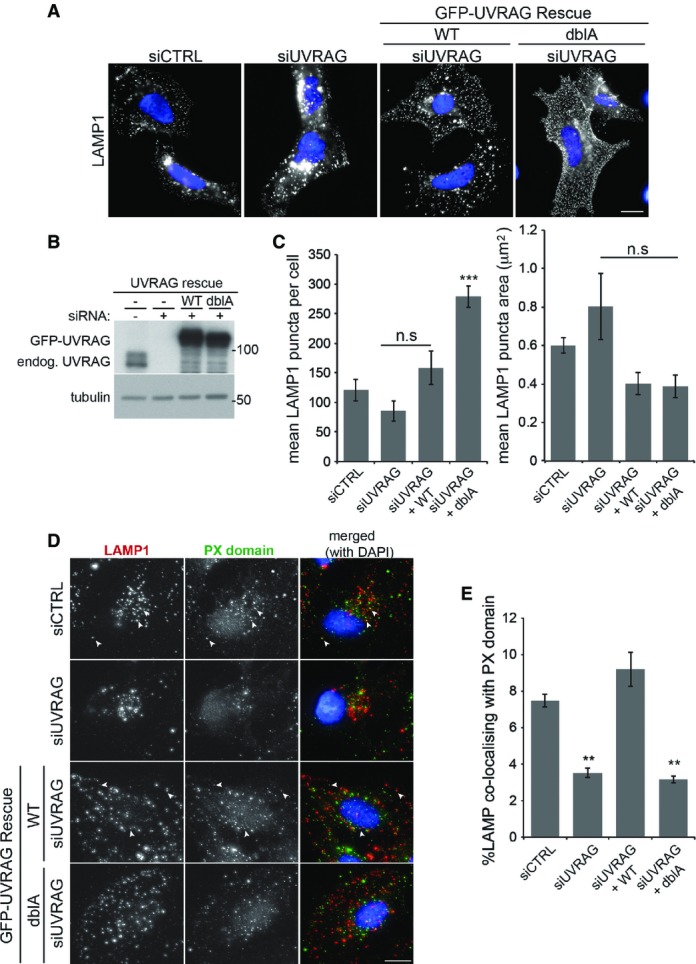
UVRAG phosphorylation alters lysosomal morphology U2OS cells or those stably expressing wild-type (WT) or S550A+S571A (dblA) GFP-UVRAG were transfected with 100 nM control or UVRAG siRNA 40 h prior to treatment. Cells were grown in complete medium prior to fixation and staining for LAMP1. Scale bar, 10 μm.

To determine whether UVRAG phosphorylation had any major effects on multiple membrane compartments, we utilised siRNA and our GFP-UVRAG rescue cells to look at cis-Golgi (GM130 staining), early endosomes (early endosomal antigen 1—EEA1) and late endosomes (cation-independent mannose-6 phosphate receptor—CI-MPR) (Supplementary Fig S4A). In fixed cells, depletion of UVRAG resulted in a dispersed and fragmented staining pattern of GM130. This is consistent with an earlier publication implicating UVRAG in Golgi-ER transport, disruption of which causes Golgi fragmentation (He *et al*, 2013). However, expression of siRNA-resistant WT or dblA GFP-UVRAG rescued this phenotype suggesting UVRAG phosphorylation does not affect Golgi integrity. Little change in EEA1 or CI-MPR staining was observed upon depletion of UVRAG or rescue with the dblA mutant, implying UVRAG phosphorylation does not play an important role in maintenance of these compartments, although we cannot rule out subtle changes at this resolution. UVRAG depletion, however, had a clear effect on LAMP1 staining (Fig6A–C). Loss of UVRAG resulted in a small decrease in LAMP1 puncta that were larger in size, similar to the effect seen with high doses of VPS34-IN1 treatment (see Fig2A), a phenotype that was rescued by expression of WT GFP-UVRAG. In contrast, rescue of the UVRAG depletion with dblA GFP-UVRAG expression resulted in a large increase in smaller dispersed LAMP1 structures (Fig6A and C). To confirm that reduction of VPS34 activity upon loss of the mTOR-UVRAG phosphorylation sites altered lysosomal PI(3)P levels, we carried out LAMP1-PX domain staining in the GFP-UVRAG rescue cells (Fig6D and E). In very good agreement with the inhibitor data shown in Fig1B and C, we found that loss of UVRAG resulted in a decrease in LAMP1-PX colocalisation, which was rescued by expression of WT GFP-UVRAG, but not the dblA mutant. We do not think that that the reduction in PX domain staining is due to mislocalisation of the dblA GFP-UVRAG mutant. Both WT and dblA GFP-UVRAG show a similar localisation with a large proportion co-localising with LAMP1-mCherry (Supplementary Fig S4B). This therefore provides additional support for the VPS34–UVRAG complex in regulating lysosomal function.

Western blot of endogenous UVRAG depletion and rescue with GFP-UVRAG as described in (A).

Quantitation of LAMP1 punctate structures and size from (A), mean ± SEM for *n* = 3 independent experiments, significance was determined by one-way ANOVA and Dunnett’s multiple comparison test to the siCTRL, ***P < 0.001 and n.s = not significant.

U2OS cells or those stably expressing wild-type (WT) or S550A+S571A (dblA) GFP-UVRAG were transfected with 100 nM control or UVRAG siRNA 40 h prior to treatment. Cells were grown in complete medium prior to fixation and staining for LAMP1 and PX domain. Scale bar, 10 μm. Arrowheads indicate co-localising punctate structures.

Quantitation of LAMP1 co-localisation with PX domain as in (D) ± SEM from *n *=* *3 independent experiments. Significance was determined by one-way ANOVA and Dunnett’s multiple comparison test to the siCTRL, **P < 0.01. Source data are available online for this figure.

**Figure 7:**
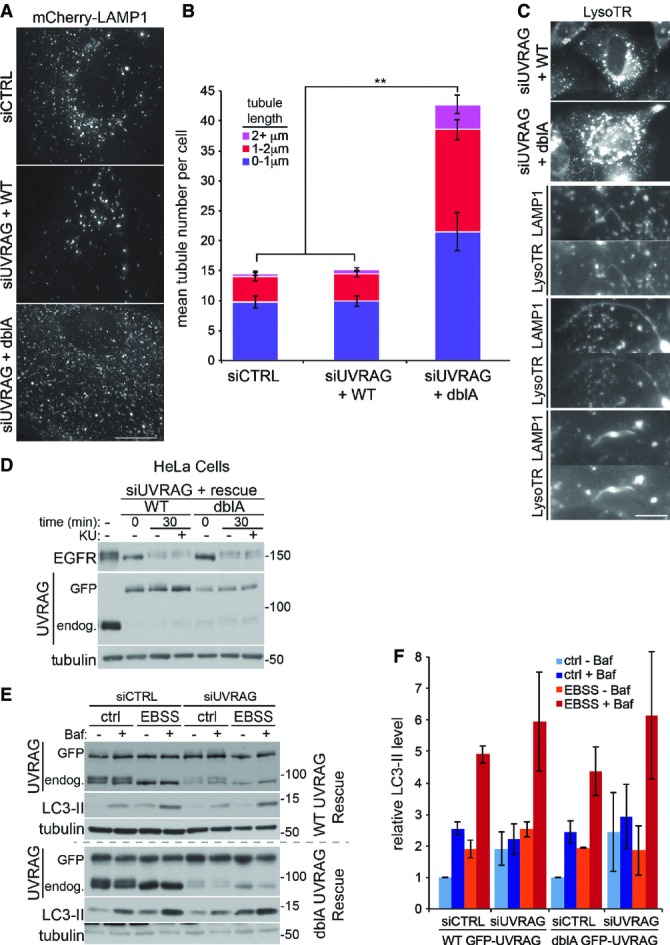
UVRAG phosphorylation regulates lysosomal tubulation U2OS cells stably expressing LAMP1-mCherry and wild-type (WT) or S550A+S571A (dblA) GFP-UVRAG were transfected with 100 nM control or UVRAG siRNA ∼40 h prior to live cell imaging in complete medium. Representative image are shown from Supplementary Movie S2A–C. Scale bar, 10 μm.

Given that LAMP1 tubules can be disrupted and fragmented by fixation, potentially giving rise to smaller structures (Sridhar *et al*, 2013), we next analysed the effect of UVRAG phosphorylation on lysosomal morphology in live cells. To monitor lysosome tubulation, we stably expressed LAMP1-mCherry in our GFP-UVRAG siRNA-rescue cell lines (Fig7A—the reader is also encouraged to look at the accompanying example movies (Supplementary Movie S2A–C), which particularly highlight the morphology). Depletion of endogenous UVRAG in cells expressing WT GFP-UVRAG showed a lysosomal morphology very similar to control cells, with a small number of tubules per cell and the majority being < 1 μm in length. However, expression of the dblA GFP-UVRAG mutant resulted in an approximate threefold increase in the total number of tubules (Fig7B). The majority of these were now twice as long as those observed in WT-expressing cells, with some extending over 10 μm in length, similar to the long tubules observed upon direct VPS34 inhibition with lower concentrations of VPS34-IN1 (compare with Fig2A and B). Interestingly, the levels of PI(3)P staining in cells expressing the dblA UVRAG mutant and in cells treated with the tubule-inducing concentration of VPS34-IN1 are approximately equal, with a twofold reduction in levels (compare Fig1A with 5C and E). Therefore, inhibition of VPS34 activity directly with VPS34-IN1 or by mutation of the mTOR phosphorylation sites results in lysosomal tubulation. We do note that mTOR inhibition blocks tubule formation, in apparent contradiction of the dblA UVRAG phospho-mutant. However, as is discussed later, this can be explained whether mTOR acts at multiple steps in tubule formation, including before the mTOR-UVRAG-VPS34 requirement.

Quantitation from (A) of mean tubules per cell and tubule length as indicated ± SEM for *n* = 3 independent experiments, significance was determined by one-way ANOVA and Dunnett’s multiple comparison test to the siCTRL, **P < 0.01.

U2OS cells stably expressing LAMP1-mCherry and WT or dblA GFP-UVRAG were transfected with 100 nM UVRAG siRNA 40 h prior to live cell imaging in complete medium and treatment with 50 nM lysotracker-648 (LysoTR). Magnified images of tubules from dblA-expressing cells are shown below. Scale bar, 5 μm.

HeLa cells or those stably expressing WT or dblA GFP-UVRAG were transfected with 100 nM control or UVRAG siRNA ∼40 h prior to treatment. Cells were serum-starved for 2 h before the addition of complete medium and 50 ng/ml EGF before lysis at 0 or 30 min in the presence or absence of 1 μM KU0063794 (KU).

MEFs stably expressing WT or dblA GFP-UVRAG were transfected with 100 nM control or UVRAG siRNA ∼40 h prior to treatment. Cells were incubated in complete medium (ctrl) or EBSS for 1 h in the presence or absence of 50 nM bafilomycin A1 (Baf).

Quantitation of normalised LC3-II from (E), mean ± SEM for *n* = 3 independent experiments. Source data are available online for this figure.

One characteristic of lysosomal tubules formed during ALR is that they lack lysotracker staining or that of resident hydrolases (Sridhar *et al*, 2013). To analyse the tubules formed in the dblA GFP-UVRAG-expressing cells, we co-stained the LAMP1-mCherry cells with lysotracker. As can be seen in Fig7C, LAMP1 structures stained with lysotracker with approximately equal intensity in either WT or dblA UVRAG-expressing cells, suggesting global lysosomal acidification was not impaired by loss of mTOR-mediated UVRAG phosphorylation. Importantly, the tubules formed in the dblA-expressing cells were still lysotracker positive. This could suggest that these tubules are different in nature to the ALR tubules or that inhibition of the mTOR-activated VPS34 impairs the selectivity mechanism, for example by simply allowing the tubules to persist attached to the originating lysosome. Data presented here showing such persistent tubules certainly support the latter conclusion.

A main question arising is what are the consequences of impaired tubule regulation for lysosomal function? VPS34 inhibition itself is well characterised in disrupting lysosomal function, primarily through blocking sorting and trafficking of receptors and hydrolases on route to lysosomes (Raiborg *et al*, 2013). However, the UVRAG phospho-mutants allow us to look more specifically at VPS34 func tion involved in lysosomal tubulation. A commonly used method to investigate lysosomal trafficking and degradation is monitoring epidermal growth factor receptor (EGFR) turnover. Upon stimulation with EGF, EGFR at the cell surface becomes activated, internalised and trafficked to the lysosome where the receptor is degraded. The majority of the tubulation experiments detailed so far were carried out in U2OS cells, and unfortunately, for this assay, we found that these cells express a relatively low level of EGFR. To overcome this assay, we switched to HeLa cells that express a much higher EGFR level (Supplementary Fig S5A). Depletion of endogenous UVRAG in cells expressing siRNA-resistant GFP-UVRAG (either WT or dblA) did not cause any noticeable change in the degradation of EGFR upon EGF stimulation (Fig7D), implying mTOR-mediated UVRAG phosphorylation does not disrupt trafficking to the lysosome or its degradative capacity. This is supported by the fact that lysosomes are still acidified in UVRAG phospho-mutant cells (Fig7C). The lack of disruption to lysosomal trafficking is supported by the observation that mTOR inhibition with KU also fails to significantly change the EGFR degradation rate (Supplementary Fig S5B and C). Similarly, it does not alter another membrane trafficking route, that of transferrin receptor recycling (Supplementary Fig S5D–F). These data suggest that mTOR activity does not play a major role in regulation of trafficking to the lysosome or general endocytic function.

A lysosomal trafficking pathway that is altered by mTOR inhibition is that of macroautophagy, being strongly induced at least in part by removal of inhibitory mTOR phosphorylation sites on the autophagy-activating ULK1 complex (Ganley *et al*, 2009; Hosokawa *et al*, 2009; Jung *et al*, 2009). The role of UVRAG in autophagy is debatable, with studies showing both a positive role (Liang *et al*, 2006) and no role (Itakura *et al*, 2008; Knævelsrud *et al*, 2010; Jiang *et al*, 2014). Given the nature of the UVRAG-mTOR-mediated phosphorylation, it would be expected not to play a role in autophagy induction, though it is possible that it may play a role in fusion and clearance of mature autophagosomes. To analyse this, we used the classical LC3 flux assay to look at the increase in LC3-II upon lysosomal inhibition (with bafilomycin A1) under normal conditions or autophagy inducing mTOR inhibition (with EBSS). We found that loss of UVRAG in the siRNA-resistant GFP-UVRAG-expressing cells, either WT or dblA, had no significant effect on LC3 flux under control conditions or short-term autophagy induction with EBSS (Fig7E and F). Thus, as expected, similar to endosome trafficking, autophagy induction and autophagosome trafficking to lysosomes do not require UVRAG phosphorylation.

### mTOR-mediated UVRAG phosphorylation enhances cell survival following long-term starvation

**Figure 8:**
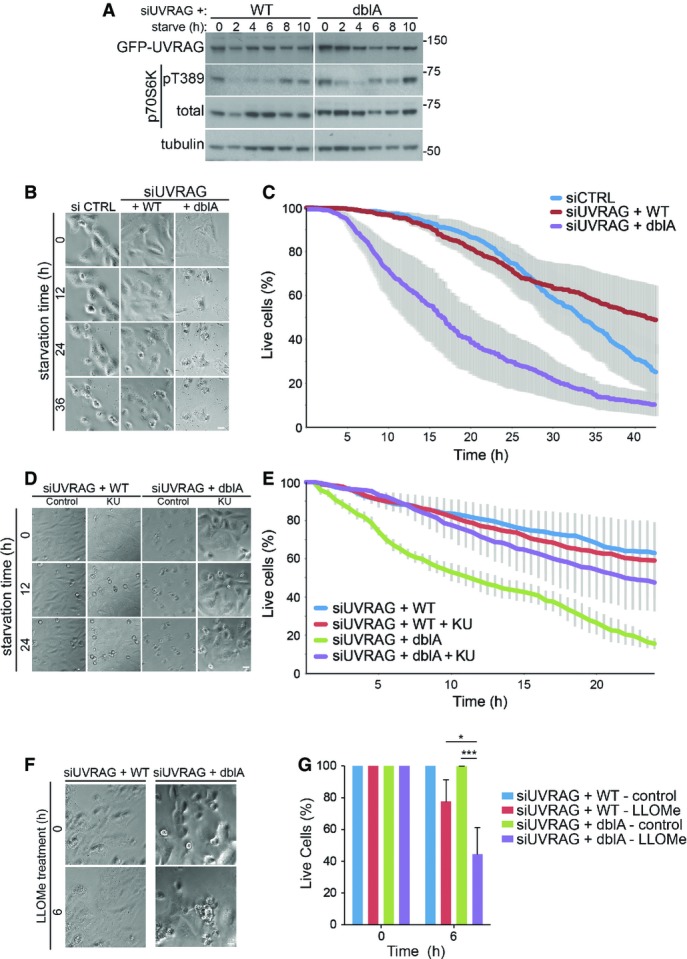
Loss of UVRAG phosphorylation impairs survival under conditions of ALR U2OS cells stably expressing wild-type (WT) or S550A+S571A (dblA) GFP-UVRAG were transfected with 100 nM UVRAG siRNA 40 h prior to treatment. Cells were serum-starved in DMEM without glutamine for up to 10 h and lysed at time points indicated.

ALR is dramatically induced only after long-term starvation (Yu *et al*, 2010); therefore, any defect in lysosome function may only manifest following longer periods of stress. A prerequisite for the induction of ALR following starvation is reactivation of mTOR as a result of increased lysosomal amino acids derived from autophagy (Yu *et al*, 2010). To look at mTOR reactivation, we used serum and glutamine starvation in cells depleted of endogenous UVRAG and expressing either siRNA-resistant WT or dblA GFP-UVRAG (Fig8A). After 2 h of starvation, mTOR activity was reduced in both WT and dblA-expressing cells, as visualised by loss of phospho-threonine 389 on p70S6K. At around 8 h of starvation, phosphorylation at the mTOR site of p70S6K started to recover in both WT and dblA cells, suggesting autophagy was able to deliver enough protein to reactivate mTOR. This is in line with our previous observation suggesting UVRAG phosphorylation is not required for autophagy. However, after 10 h of starvation we observed significant cell death only in dblA UVRAG-expressing cells. To analyse this further, we used time-lapse microscopy to monitor cell death of UVRAG-expressing cells (Fig8B and C). Under the starvation conditions, the majority of cells died and displayed an apoptotic phenotype with extensive membrane blebbing followed by leakage of cytosolic contents. Control-depleted cells and UVRAG-depleted cells expressing WT GFP-UVRAG were very similar in their rates of cell death that increased gradually following approximately 20 h of starvation. What was striking was that cells depleted of endogenous UVRAG and expressing the dblA mutant showed a much greater sensitivity to starvation, with a rapid induction of cell death following only 8 h of treatment. This timing correlates very well with the reactivation of mTOR in these cells (compare with Fig8A). As the dblA-expressing cells have a defect in lysosomal tubulation, it is possible that this acts as a trigger for cell death under prolonged starvation. Given that mTOR inhibition prevents tubule formation (Fig2A and B), we asked whether this would reverse the increased cell death observed in the dblA cells. Treatment of WT and dblA cells, which had endogenous UVRAG depleted by siRNA, with KU mTOR inhibitor did indeed increase the survival of the dblA cells under starvation, while having little effect on the WT GFP-UVRAG-expressing cells (Fig8D and E). This suggests the increased tubulation is responsible for the increased cell death. We do not yet know the mechanism whereby prolonged tubulation causes cell death, but it is tempting to speculate that lysosome integrity is impaired and increased stress leads to lysosomal membrane permeabilisation and release of hydrolases into the cytosol. In support of this, we found that dblA-expressing cells were much more sensitive to lysosomal damage caused by the lysosomotropic reagent L-leucyl-L-leucine methyl ester (LLOMe, Maejima *et al*, 2013). LLOMe treatment for 6 h with our siRNA-rescue cells resulted in over a twofold increase in cell death in dblA cells compared to WT UVRAG cells (Fig8F). We do note however that the cell death mechanism may be independent of lysosomal tubules, especially given that UVRAG has been implicated in DNA repair (Zhao *et al*, 2012). However, based on this study, we found no difference in WT vs. dblA UVRAG interaction with the Ku70-DNA-PK complex (Supplementary Fig S6A). Neither did we see any increase in DNA damage in WT vs. dblA-expressing cells, as indicated by γ-H2AX staining under normal or starvation conditions as well as UV-induced DNA damage (Supplementary Fig S6B and C). Taken together, the data currently suggest that persistent lysosomal tubulation, caused by loss of mTOR-mediated UVRAG phosphorylation, severely impedes cell survival and that the regeneration of lysosomes following starvation is critical to this process.

Cells were grown in glutamine-free DMEM and monitored by bright-field microscopy with images captured every 10 min up to 42 h. Scale bar, 10 μm.

Quantitation of (B) represents mean % live cells relative to time ± SEM for *n* = 3 independent experiments.

Cells were grown in serum and glutamine-free DMEM for 12 h prior to the addition of DMSO or 1 μM KU and monitored by bright-field microscopy. Scale bar, 10 μm.

Quantitation of (D) represents mean % live cells relative to time ± SEM for *n *=* *3 independent experiments.

Cells were grown in complete medium in the presence or absence of 1 mM LLOMe and monitored by bright-field microscopy. Scale bar, 10 μm.

Quantitation of (F) represents mean % live cells relative to time ± SEM for *n *=* *3 independent experiments. Significance was determined by two-way ANOVA and Bonferroni post-test, *P < 0.05 and ***P < 0.001. Source data are available online for this figure.

## Discussion

The data presented here uncover a direct role for VPS34 lipid kinase activity in regulating lysosomal or, more specifically autolysosomal, tubulation. Two strong lines of evidence led to this conclusion. Firstly, pharmacological inhibition of VPS34 using limiting concentrations of VPS34-IN1, which was sufficient to deplete an identified lysosomal pool of PI(3)P, resulted in a significant increase in lysosomal tubules. Secondly, loss of newly discovered mTOR phosphorylation sites on UVRAG that caused a reduction in VPS34 activity also led to a large increase in lysosomal tubules. The observation that trafficking prior to the lysosome appeared unaffected by the loss of UVRAG phosphorylation implies that the lysosome itself is the major site for this form of regulation. Indeed, this is where active mTOR is localised (Sancak *et al*, 2010; Betz & Hall, 2013), and as we now show here, UVRAG too.

mTOR and VPS34 have a complex relationship, and multiple studies have shown that nutrient starvation, and inhibition of mTOR, reduce total cellular PI(3)P levels (Byfield *et al*, 2005; Russell *et al*, 2013; Yuan *et al*, 2013). However, this is not so simple as it first seems as there is more than one cellular pool of PI(3)P, such as that present at early endosomes, autophagosomes or as identified here, lysosomes. These pools can be differentially regulated, in part due to the different complexes of VPS34. This is most striking upon inhibition of mTOR and autophagy induction, where the autophagic pool of PI(3)P is dramatically increased, in part through phosphorylation/dephosphorylation of the VPS34–BECLIN1–ATG14L complex by AMPK (Kim *et al*, 2013), ULK1 (Russell *et al*, 2013) and mTOR (Yuan *et al*, 2013) kinases. This is in contrast to the endocytic and newly identified lysosomal pools that are decreased, which we now show is due to dephosphorylation of the distinct VPS34–BECLIN1–UVRAG complex. Taken together, this suggests that VPS34 and its associated complexes act as signalling nodes to drive and co-ordinate multiple trafficking pathways. We do note that during the submission of this manuscript, a paper was published describing mTOR phosphorylation of UVRAG at serine 498 (S498) (Kim *et al*, 2015) However, phosphorylation inhibited VPS34 activity through enhanced RUBICON binding and decreased trafficking of endosomes and autophagosomes to the lysosome. This is somewhat at odds with the data presented here. We did identify S498 of UVRAG as being phosphorylated in cells; however, unlike S550 and S571, this was not affected by mTOR inhibition and mutation of this site did not reduce endogenous mTORC1 phosphorylation of UVRAG *in vitro* (Supplementary Fig S3). Similarly, mTOR inhibition by treatment with KU or EBSS did not alter the binding of RUBICON to the VPS34 complex (Fig5G). We also found that mTOR inhibition reduced VPS34 activity, which is consistent with the previously published work from other groups (Byfield *et al*, 2005; Russell *et al*, 2013; Yuan *et al*, 2013). We cannot fully explain these discrepancies, but an obvious explanation is that in our cellular systems at least, another kinase is responsible for UVRAG phosphorylation at S498.

The autolysosomal tubulation observed here differs somewhat for the original observations of ALR (Yu *et al*, 2010). Firstly, we find tubulation occurs under normal nutrient-rich growth conditions. We take this to mean that ALR is constantly occurring, but under these basal conditions, it is not as evident as those following longer-term starvation. The fact that we are blocking this process by VPS34 inhibition allows us to more easily visualise it here. Secondly, the classical ALR tubules are not positive for lysotracker or cargo such as LC3, whereas the tubules described here are positive for both. A potential explanation for this may partially due to the persistent nature of the tubules: their prolonged existence may mean it is more likely that the quality control mechanisms to prevent passage of cargo into tubules are overcome. Further work will be needed to clarify this situation.

mTOR activity has previously been shown to regulate lysosomal tubulation, being essential for the process of ALR, through an unknown mechanism. Our study now provides a further link in showing that mTOR is needed not only for tubule initiation, but also for tubule maintenance by phosphorylating UVRAG and activating VPS34. We do not yet know the exact function of PI(3)P in regulating lysosomal tubules, though given the persistent and extended phenotype upon VPS34 inhibition, it is tempting to suggest a role in tubule scission from the donor lysosome. Dynamin 2 has been shown to play an important role in tubule scission at lysosomes, and the use of the dynamin inhibitor dynasore rapidly induces a similar LAMP1-positive tubular phenotype (Schulze *et al*, 2013). Dynamin activity has been shown to be activated by multiple phosphoinositides, including PI(3)P, PI(4)P and PI(4,5)P_2_, *in vitro* (Yarar *et al*, 2008). Interestingly, this same study showed that the PX domain containing protein SNX9 synergised with PI(3)P and other phosphoinositides to stimulate dynamin activity further. We are currently investigating whether these proteins are involved in the tubulation observed here.

Another candidate PI(3)P-binding protein that may regulate lysosomal tubulation is SPASTIZIN/SPG15/FYVE-CENT, the gene of which is frequently mutated hereditary spastic paraplegia (HSP) (Hanein *et al*, 2008). Work on zebrafish oocytes showed that SPASTIZIN is required for vesicle scission from lysosome-related cortical granules, potentially by regulating dynamin (Kanagaraj *et al*, 2014). Very recently in human cells, SPASTIZIN was shown to be important for ALR (Chang *et al*, 2014). This study showed that the FYVE domain of SPASTIZIN is important for its lysosomal localisation, which strongly supports our data showing PI(3)P at the lysosome. The study also found that loss of SPASTIZIN impaired lysosomal tubulation, which is in contrast to our study that showed persistent tubules upon VPS34 inhibition. However, it was not clear from this study whether the block in tubulation is due to the PI(3)P-binding properties of the protein. It is also interesting to note that lysosomes were still functional upon loss of SPASTIZIN, in agreement with our results in cells expressing the dblA UVRAG mutant.

Phosphoinositides play an important role in lysosomal tubulation, and not just through the potential interaction with dynamin as mentioned above. Two important studies showed that PI(4)P and PI(4,5)P_2_ play essential roles in controlling tubule initiation. The first study showed that PI(4,5)P_2_, produced by PIP5K1B at lysosomal budding sites, led to the recruitment of the clathrin machinery to enable tubule formation (Rong *et al*, 2012). The same study also showed that the related kinase, PIP5K1A, localised to the leading edges of tubules and the produced PI(4,5)P_2_ aided in tubule scission. The second study showed that PI4KIIIβ, a lipid kinase responsible for PI(4)P production, is also required to allow controlled tubule production (Sridhar *et al*, 2013). This is not only by providing PI(4)P, the substrate for the PIP5Ks to make PI(4,5)P_2_, but also to prevent uncontrolled tubulation that occurred upon loss of PI4KIIIβ. The enhanced formation of lysosomal tubules upon loss of PI4KIIIβ is somewhat similar to the tubules formed upon loss of VPS34 activity described here, implying that lysosomal PI(3)P could be involved in preventing tubule formation rather than controlling scission from lysosomes. However, the tubules formed upon loss of VPS34 activity differ somewhat from those produced by loss of PI4KIIIβ. The VPS34-dependent tubules stain with lysotracker (see Fig6C), while the PI4KIIIβ-dependent tubules do not. In fixed cells, loss of VPS34 activity through the UVRAG phospho-mutant results in an increased number of small, evenly distributed LAMP1 puncta (see Fig5A), while loss of PI4KIIIβ shows a clustered pattern of large LAMP1 structures.

**Figure 9:**
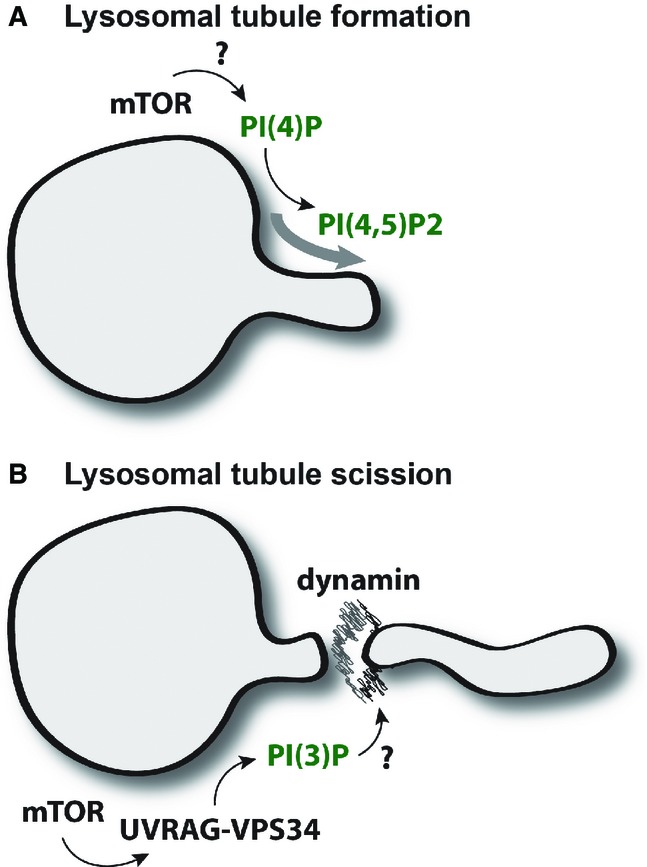
Model of PI(3)P-mediated lysosomal regulation Autophagic lysosomal regulation (ALR) and tubulation is driven by the conversion of PI(4)P to PI(4,5)P_2_ but is fundamentally dependent upon mTOR activity for initiation via unknown mechanisms.

Having three different forms of phosphoinositide may seem an excessive requirement for controlling lysosomal tubulation. However, having such a requirement for different modifications of a single inositide lipid allows for rapid control of signalling and maturation of the tubulation process. Interestingly, PI(3)P and PI(4,5)P2 have been shown to co-operate to drive actin assembly and it is possible this kind of regulation could be happening during lysosomal tubulation (Gallop *et al*, 2013). A simple model for the roles of these lipids is summarised in Fig9. Clearly, more work is needed to fully characterise and understand the relationships between these three different phosphoinositides and the mechanisms that coordinate their kinases and phosphatases. Regardless, the process of tubulation appears to be essential for normal cell function. Though ALR has been characterised, it has been difficult to show the specific consequences that disrupting the process has on cell survival. The identification of a phospho-mutant of UVRAG that alters lysosomal tubulation, yet does not significantly affect endocytic trafficking, has allowed us to look into this in more detail. The increased rate of cell death upon starvation in the dblA UVRAG cells does indeed suggest ALR tubulation is essential in maintaining lysosomal function and cell survival, especially as this can be reversed by mTOR inhibition. Given the recent SPASTIZIN link to ALR (Chang *et al*, 2014), the results presented here suggest increased rates of cell death could be responsible for the degeneration of motorneurons in HSP. Though the mechanisms of cell death appear to have the hallmarks of apoptosis, we do not yet know how it is triggered; though with the increased susceptibility of the dblA UVRAG mutant cells to lysosomal damage, it is tempting to speculate that lysosomal integrity is impaired leading to the release of hydrolases into the cytosol.

mTOR acts at a secondary step via phosphorylation of UVRAG to increase formation of lysosomal PI(3)P that appears to be critical for tubule scission, a process regulated by the GTPase dynamin.

The work presented here not only shows that the nutrient-sensing mTOR protein kinase directly activates the membrane trafficking VPS34 lipid kinase, but that this activation is important in regulating lysosomal tubulation and survival during starvation. Given the link between lysosomes and cell survival with many diseases such as neurodegeneration and cancer, further analysis of this pathway may lead to future therapeutic approaches.

## Materials and Methods

### Materials

Antibodies (with catalogue number) against 4E-BP1 (9452), DNA-PKc (4602), EEA1 (2411), GM130 (2296), γ-H2AX (2577), mTOR (2983), p70S6K (2708), p70S6K P-T389 (9205), RUBICON (8465), α-tubulin (2125) and ULK1 P-S757 (6888) were from Cell Signaling Technologies. GST-HRP (Ab3461) and CI-MPR (ab2733) were from Abcam. GFP (11814460001) was from Roche. FLAG-HRP (A8592), LAMTOR1 (HPA002997) and ULK1 (A7481) were from Sigma-Aldrich. EGFR (sc-03), CD63 (sc-5275) and LAMP1 (sc-20011) were from Santa Cruz Biotechnology, Inc. ATG14L (PD026) and Beclin1 (PD017) were from MBL International Corporation. Sheep anti-UVRAG was generated against full-length human GST-UVRAG and affinity-purified by the Division of Signal Transduction Therapy (DSTT), University of Dundee. Phospho-specific antibodies against UVRAG S550 and S571 were also generated in sheep and affinity-purified by the DSTT. The following peptides were used for immunisation: S550—KITSLSSS*LDTSLD and S571—KKGEDLVGS*LNGGHANRR.

All cDNA constructs and antibodies against LC3, RAPTOR, RICTOR, UVRAG, UVRAG P-S550, UVRAG P-S571 and VPS34 were generated by the DSTT. All recombinant proteins, plasmids and antibodies generated for the present study are available upon request and are described in further detail on our reagents website (https://mrcppureagents.dundee.ac.uk/).

Alexa Fluor-594 labelling kit (A30008), ProLong gold antifade mountant with DAPI (P36931), lysotracker-648 (L12492) and transferrin-594 (T-13343) were from Life Technologies. Bafilomycin A1 was purchased from Enzo and KU0063794 from Tocris. Leu-leu methyl ester hydrobromide (LLOMe) was purchased from Sigma.

### Cell culture and lysis

U2OS, HeLa, mouse embryonic fibroblasts (MEFs) and HEK293 cells were maintained in a complete medium of DMEM (Gibco) supplemented with 10% foetal bovine serum (FBS, Hyclone), 2 mM L-glutamine (Lonza) and 1% penicillin and streptomycin (Lonza). Cells were cultured within a humidified environment at 37°C with 5% CO_2_. Wild-type and knockout RICTOR MEFs (Shiota *et al*, 2006) were provided by Prof. M. Magnuson (Vanderbilt University School of Medicine, USA).

For lysis, cells were washed twice on ice with PBS and subsequently scraped with ice-cold NP-40 lysis buffer (50 mM Hepes pH 7.4, 150 mM NaCl, 1 mM EDTA, 10% (v/v) glycerol, 0.5% NP-40) unless otherwise stated. All lysis buffers were supplemented with 1 mM DTT, 1 mM PMSF and phosphatase inhibitors. Samples were clarified by centrifugation at 20,000 *g* for 10 min at 4°C and protein concentration determined by Bradford (500-0006, Bio-Rad protein assay).

### Transfections

Stable cell lines were generated by retroviral transduction. The pBabe construct of interest was co-transfected into 293FT cells with GAG/POL and VSV-G expression plasmids (Clontech) for retrovirus production using Lipofectamine 2000 (Life Technologies) as per the manufacturer’s instructions. Virus was harvested 48 h post-transfection, passed through a 0.45-μm filter and added to target cells in the presence of 10 μg/ml polybrene (Sigma). After 24 h, cells were selected by addition of 10 μg/ml puromycin (Sigma) and utilised for experiments following complete death of non-transfected controls.

For knockdown of endogenous UVRAG, cells were transiently transfected with a previously described siRNA—GGACAAAGGAAGTGCATTT (Liang *et al*, 2006). siRNA was transfected using Transfectin (Bio-Rad) as per the manufacturer’s protocol, and cells were typically utilised for experiments ∼40 h post-transfection.

### PI(3)P PX domain staining

For selective PI(3)P staining, the GST-tagged PX domain (residues 1–148 of p40^phox^) was expressed in *E. coli* (BL21) and purified over a glutathione column using standard procedures. The recombinant protein was chemically conjugated to Alexa Fluor-594 as per the manufacturer’s protocol.

For staining, following treatment cells were washed once on ice with phosphate-buffered saline (PBS) and glutamate buffer (25 mM Hepes pH 7.4, 25 mM KCl, 2.5 mM Mg-acetate, 5 mM EGTA, 150 mM K-glutamate). Coverslips were snap-frozen in liquid N_2_ and thawed prior to two further washes with ice-cold glutamate buffer. Cells were fixed with 3.7% (w/v) formaldehyde, 200 mM Hepes pH 7.4 for 30 min at RT and subsequently quenched by two washes and incubation for 10 min in 10 mM Hepes pH 7.4, DMEM. Samples were blocked by washing twice and incubated for 30 min in blocking buffer (1% (w/v) BSA, PBS). Coverslips were incubated for 1 h at RT with 5 μg/ml PX domain conjugate and washed three times in blocking buffer. Coverslips were washed once more in ddH_2_O prior to mounting with ProLong Gold antifade mountant.

### Electron microscopy

Immuno-EM was carried out on cells fixed in 1% glutaraldehyde in 0.2 M PIPES buffer pH 7.2. After pelleting and cryoprotection in 2.1 M sucrose in PBS, ultra-thin frozen sections were cut at −100°C (EM FC7 ultracryomicrotome; Leica, Vienna, Austria). Labelling for LAMP1 was performed at room temperature followed by secondary rabbit anti-mouse (Southern Biotech). Labelling for PI(3)P was at low temperature (Watt *et al*, 2002) using the GST-PX domain followed by antibodies against GST (Abcam, cat no 9085). Molecular contrasting was completed using 10 nm protein A gold from BBI, and structure contrasting was achieved using uranyl acetate-methyl cellulose (according to Griffiths *et al*, 1984). After air-drying, the sections were imaged using a JEOL 1200- transmission electron microscope at 80 kV and images taken with a Gatan Orius 200 digital camera (Gatan, Abingdon Oxon).

### Live cell imaging

For live cell imaging experiments, cells were seeded onto glass-bottom dishes (627870, Greiner Bio One) a minimum of 16 h prior imaging. Treatments were carried out in phenol red-free DMEM buffered with 20 mM Hepes pH 7.4, and imaging was carried out by widefield deconvolution microscopy (Deltavision Elite, GE Healthcare) in a heated environment chamber. Images were acquired and deconvolved using softWoRx 5.0 (Applied Precision, GE Healthcare).

For long-term starvation treatments, cells were maintained in a humidified environmental chamber with 5% CO_2_ and imaged by bright-field (Eclipse TiE, Nikon Instruments). Images were acquired and analysed utilising NIS-Elements (Nikon Instruments).

### mTORC1 *in vitro* kinase assay

HEK293 cells were grown in complete medium and stimulated with 50 ng/ml IGF1 for 30 min to hyper-activate mTOR prior to lysis in CHAPS buffer (20 mM Tris pH 7.4, 137 mM NaCl, 2 mM EDTA, 10% (v/v) glycerol, 2% (w/v) CHAPS). Endogenous RAPTOR was immunoprecipitated with Protein G Sepharose and washed twice with CHAPS buffer + NaCl (0.5 M total), twice with CHAPS buffer and twice with kinase buffer (25 mM Hepes pH 7.4, 50 mM KCl) and left with ∼10 μl buffer on the beads. Per reaction, 1 μg substrate was added in 20 μl kinase buffer prior to the addition of 10 μl ATP mix (40 mM MnCl_2_, 80 μM ATP, 1 μCi ^32^P γ-ATP in kinase buffer). Reactions were agitated at 30°C for 30 min and terminated by addition of 10 μl 5× sample buffer (300 mM Tris pH 6.8, 8% (w/v) SDS, 50% (v/v) glycerol, 0.025% (w/v) bromophenol blue).

Samples were separated by SDS–PAGE and transferred to PVDF membrane before detection of ^32^P incorporation by autoradiography.

### Mass spectrometry

Samples were resolved by SDS–PAGE and Coomassie staining (ISB1L, Expedeon), and bands were excised and processed for mass spectrometry. Samples were alkylated in gel with iodoacetamide (Sigma) and digested overnight with trypsin gold (V5280, Promega). Peptides were extracted and analysed by LC-MS/MS using an Orbitrap Classic mass spectrometer (Thermo Scientific). Proteins and phosphorylation sites were identified from peptides by Mascot software (Matrixscience). Phosphorylation sites were represented by comparison of the extracted ion chromatograms between samples in Xcalibur (Thermo).

### VPS34 *in vitro* kinase assay

Cells were treated as described in figure legends prior to lysis in NP-40 lysis buffer. Endogenous or exogenous UVRAG was immunoprecipitated, and beads were washed twice in NP-40 lysis buffer + 0.5 M NaCl (final), twice with NP-40 lysis buffer and finally twice with lipid kinase assay (LKA) buffer (10 mM MnCl_2_, 20 mM Tris pH 7.5, 67 mM NaCl, 0.02% (w/v) CHAPS).

Associated VPS34 was assayed *in vitro* with a final concentration of 10 μg phosphatidylinositol (PI) liposomes (bovine liver PI, extruded through a 100-nm filter—Avanti Mini-Extruder), 5 μM ATP and 7.5 μCi ^32^P γ-ATP in LKA buffer. Reactions were agitated for 30 min at 30°C before centrifugation through a Spin-X column to isolate beads, and the addition of 500 μl methanol:chloroform:hydrocholoric acid (200:100:3.5) terminated the reaction. 1× sample buffer (50 mM Tris pH 6.8, 2% (w/v) SDS, 10% glycerol (v/v), 0.005% bromophenol blue) was added to the beads removed by Spin-X and analysed by Western blot as a control for VPS34 levels. A total of 180 μl of chloroform and 300 μl of 0.1 M hydrochloric acid were added to the solvent of each sample and centrifuged at 1,000 *g* for 1 min at RT to phase-split components. The lower lipid-containing chloroform layer was retained and dried by centrifugal evaporation. Lipids were re-dissolved in chloroform and spotted onto thin layer chromatography (Silica 60, Merck-Millipore) activated in potassium oxalate (1% (w/v) potassium oxalate, 5 mM EDTA, 50% (v/v) methanol). Plates were ran in a solvent system comprised of methanol:chloroform:water:ammonium hydroxide (47:60:11.2:2) to separate phosphoinositol products. Once dried, the incorporation of ^32^P was quantified by phosphorimager (FLA-2000, Fujifilm) and AIDA image analyser (Raytest). Values were normalised to VPS34 level determined by Western blot.

### Laser microirradiation

Cells were grown on glass-bottom dishes in complete medium and in the presence of 10 μM BrdU for 48 h. Laser micro-irradiation was carried across the nuclei in 100 cells per condition with a 405-nm laser attached to a PALM microscope (Zeiss). Cells were then returned to 37°C for 1 h prior to fixation with 3.7% (w/v) formaldehyde, 200 mM Hepes pH 7.4 and then immunofluorescence staining for GFP and γ-H2AX to visualise laser-induced damage.

### Quantitation

Western blots and mTOR kinase assays were quantified by densitometry using ImageJ. Immunofluorescence images were quantified for cell intensity, punctate structures and co-localisation utilising NIS-Elements. Automated counting was carried out by intensity thresholding, and the criteria set was kept consistent within experiments between conditions and checked thoroughly to ensure accurate representation of the data. Typically, 5–10 fields of view were analysed per condition (50+ cells minimum) per experimental replicate. LAMP1 tubule length and number were analysed by ImageJ. For tubule analysis, typically 5–10 cells per condition were quantified per experimental replicate.

### Statistics

Statistical analysis and *post hoc* tests were carried out as described in figure legends using GraphPad Prism v5.0. The statistical significance is denoted on graphs by asterisks (*), where *P < 0.05, **P < 0.01, ***P < 0.001 and n.s. = not significant.

## Supporting Information

**Source Data for Supplementary Figure S6**

**Review Process File**
