# Supplementary figures and images for: mTOR activates the VPS34–UVRAG complex to regulate autolysosomal tubulation and cell survival
Source: EMBO J. 2015 Jul 2;34(17):2272–90. doi: 10.15252/embj.201590992 (PMC4585463; doi:10.15252/embj.201590992)

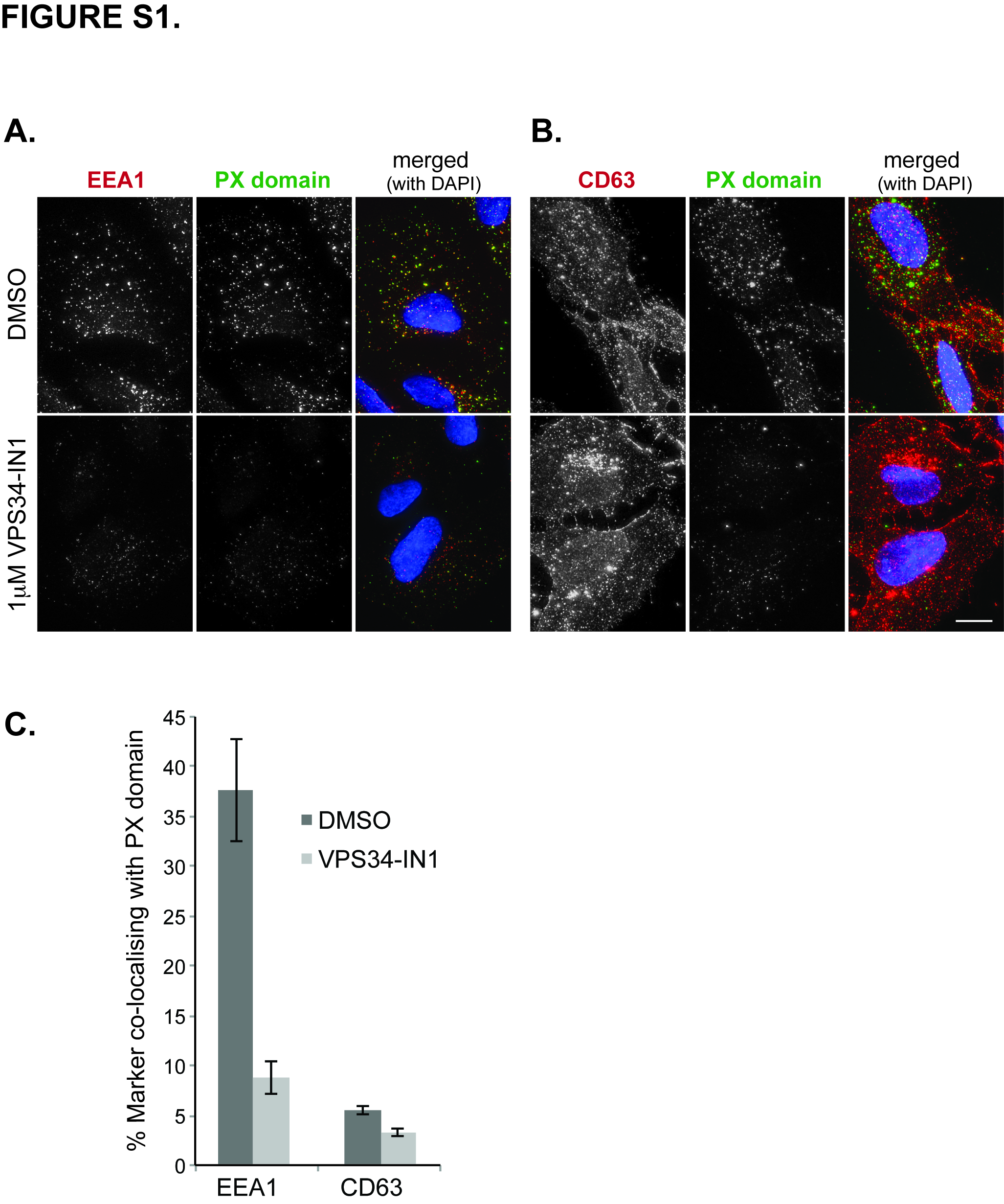

Supplement: Supplementary file 1 [file embj0034-2272-sd1.tif]

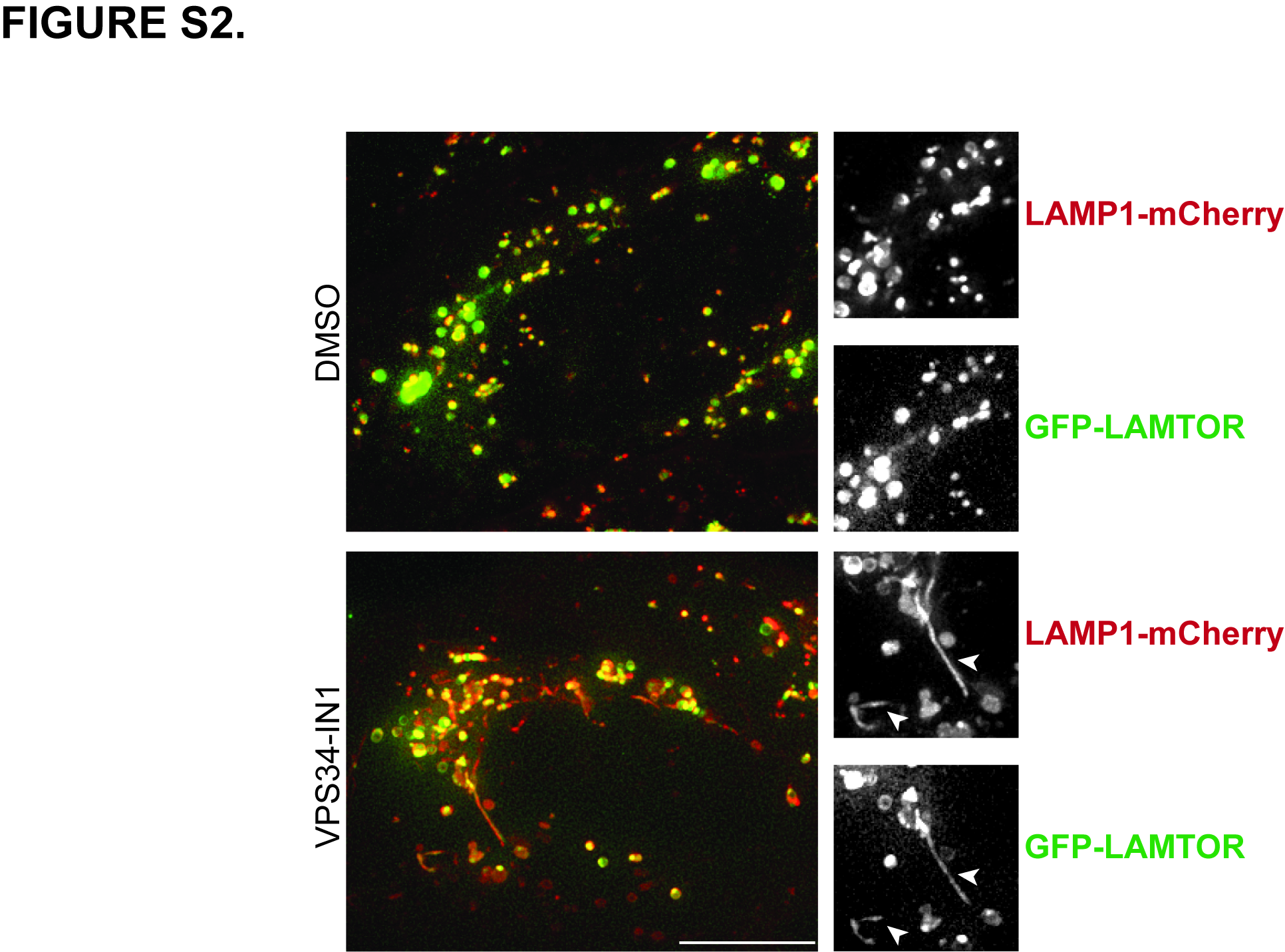

Supplement: Supplementary file 2 [file embj0034-2272-sd2.tif]

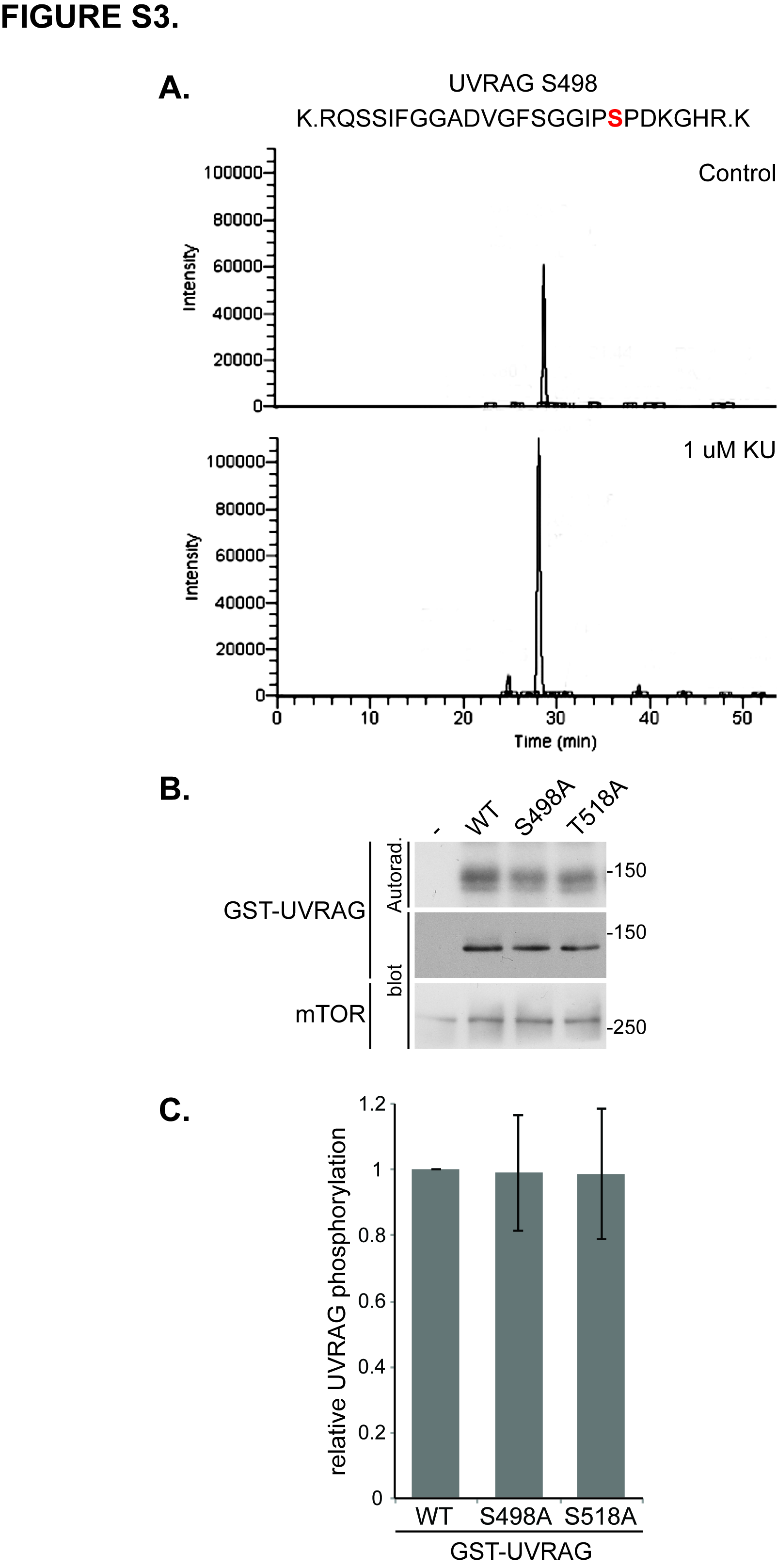

Supplement: Supplementary file 3 [file embj0034-2272-sd3.tif]

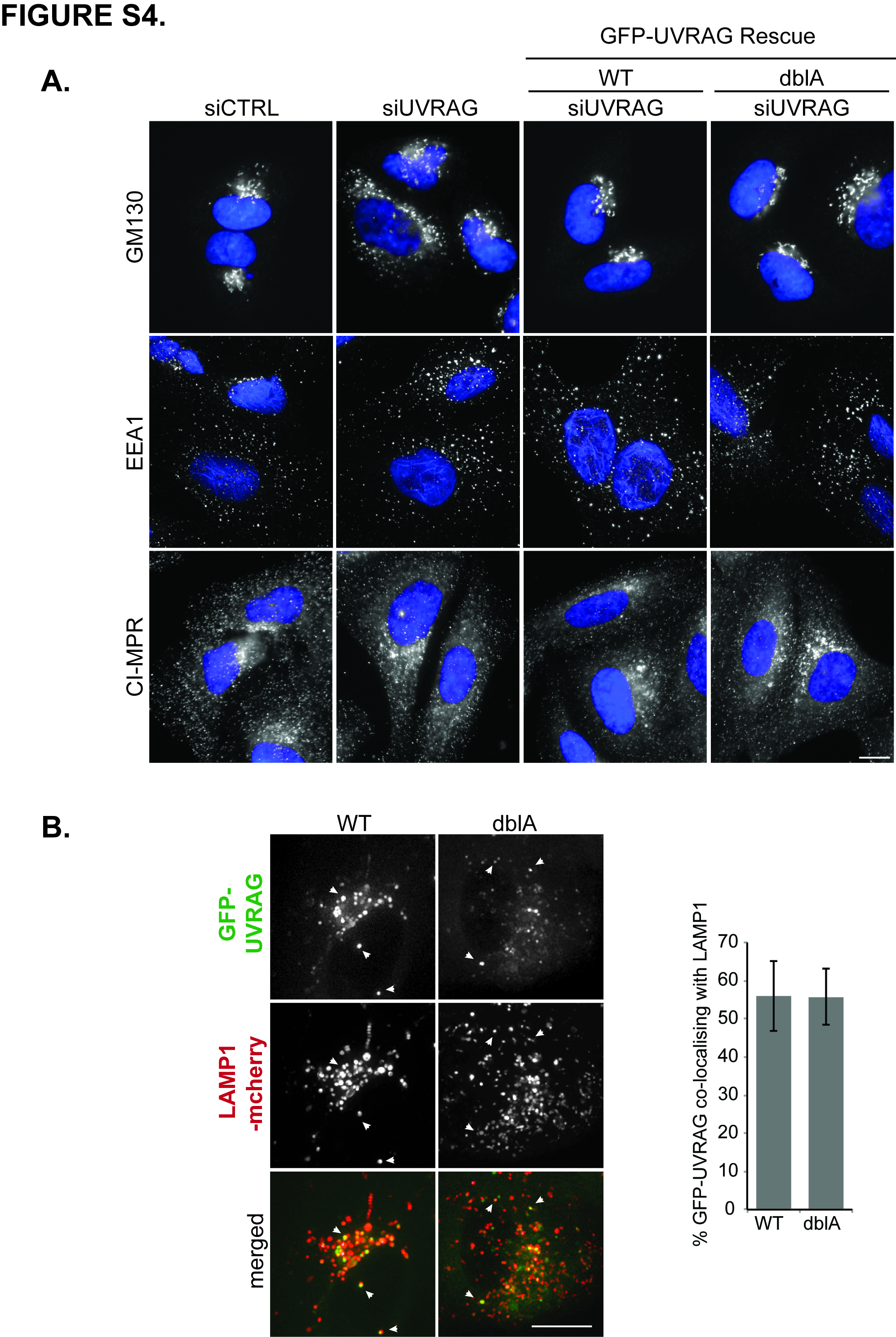

Supplement: Supplementary file 4 [file embj0034-2272-sd4.tif]

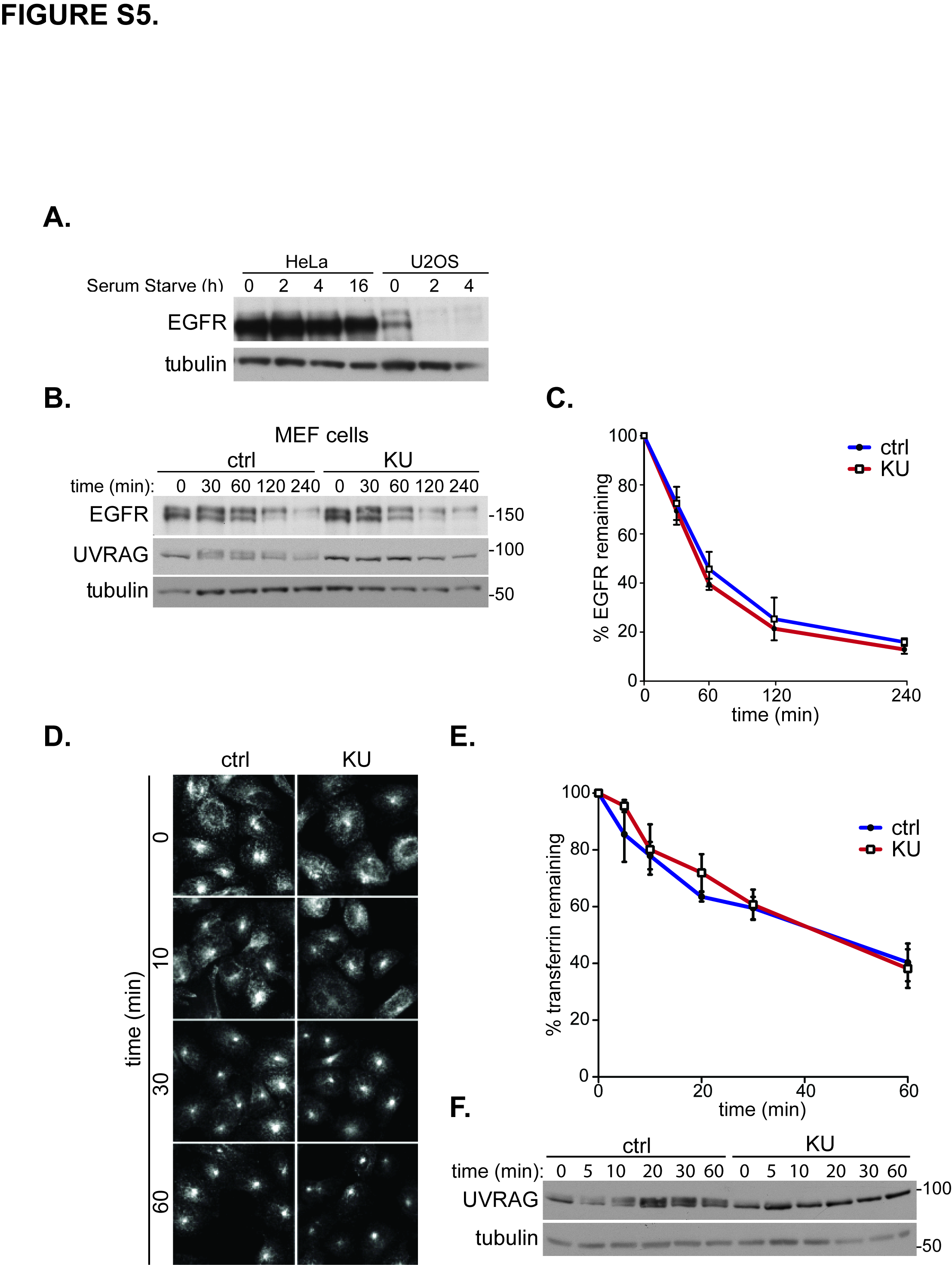

Supplement: Supplementary file 5 [file embj0034-2272-sd5.tif]

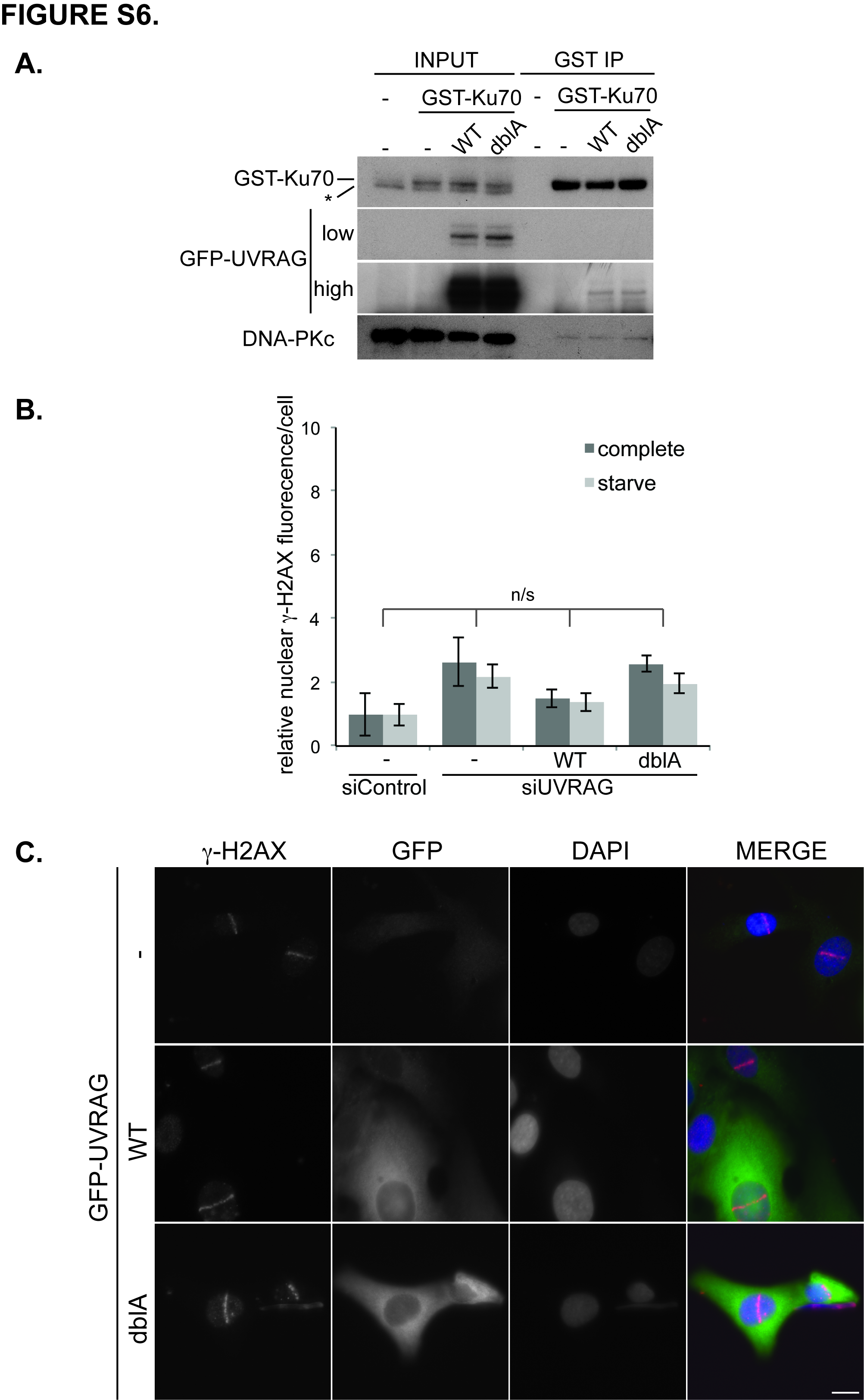

Supplement: Supplementary file 6 [file embj0034-2272-sd6.tif]

Figure S3 - Source Data

B.

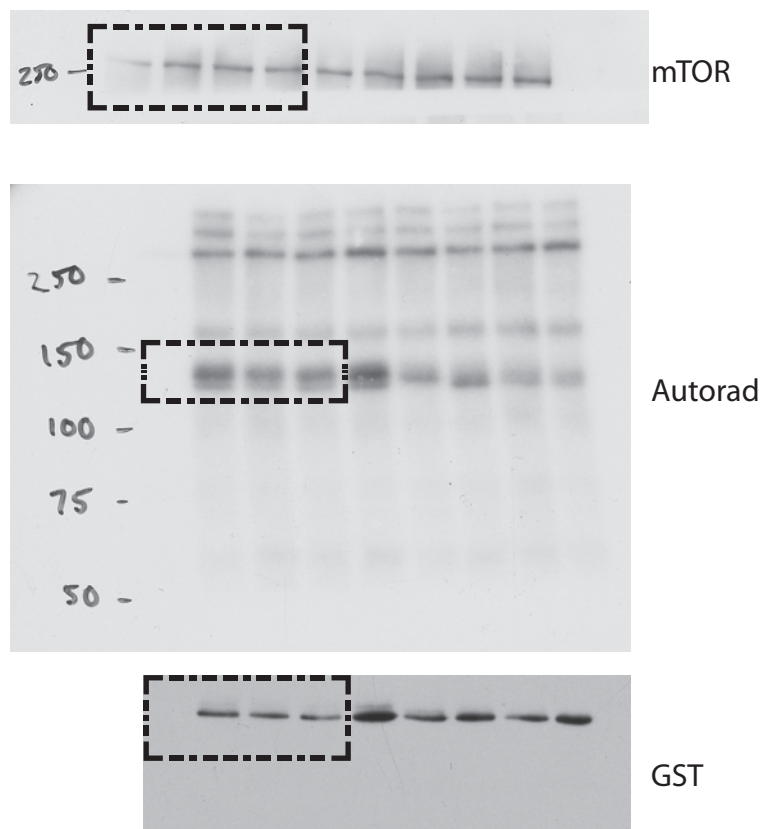

Supplement: Supplementary file 10 [file embj0034-2272-sd10.pdf]

Figure S5 - Source Data

A.

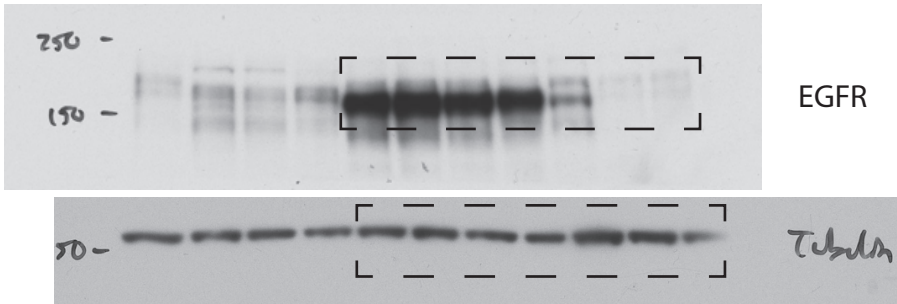

B.

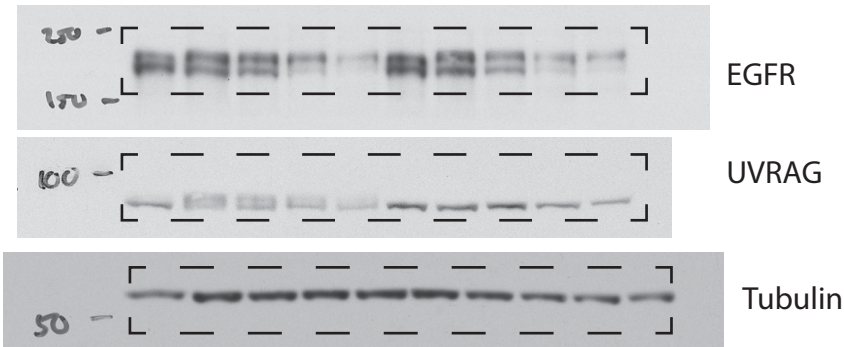

F.

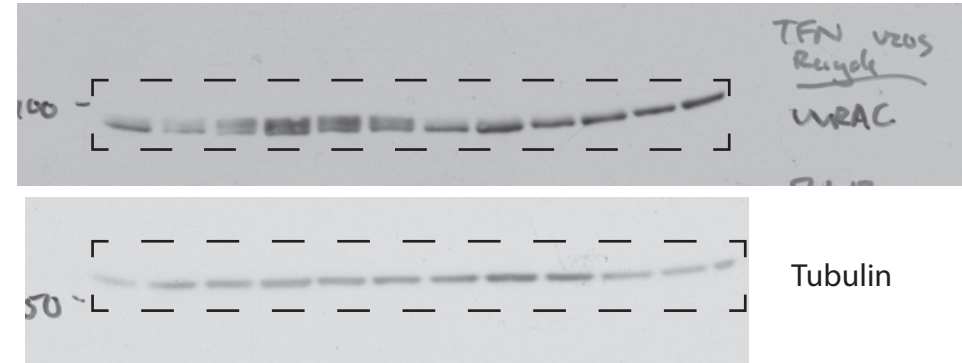

Supplement: Supplementary file 11 [file embj0034-2272-sd11.pdf]

Figure S6 - Source Data

A.

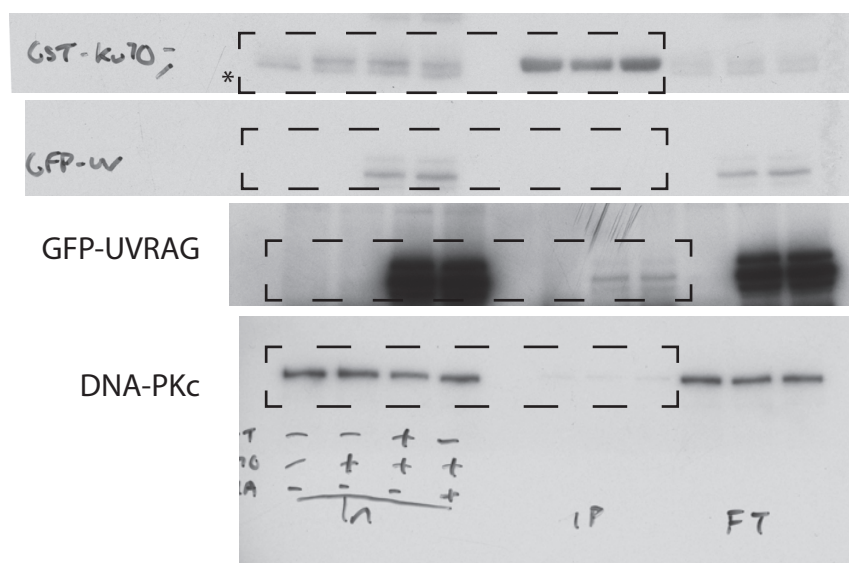

Supplement: Supplementary file 12 [file embj0034-2272-sd12.pdf]

Figure 3 - Source Data

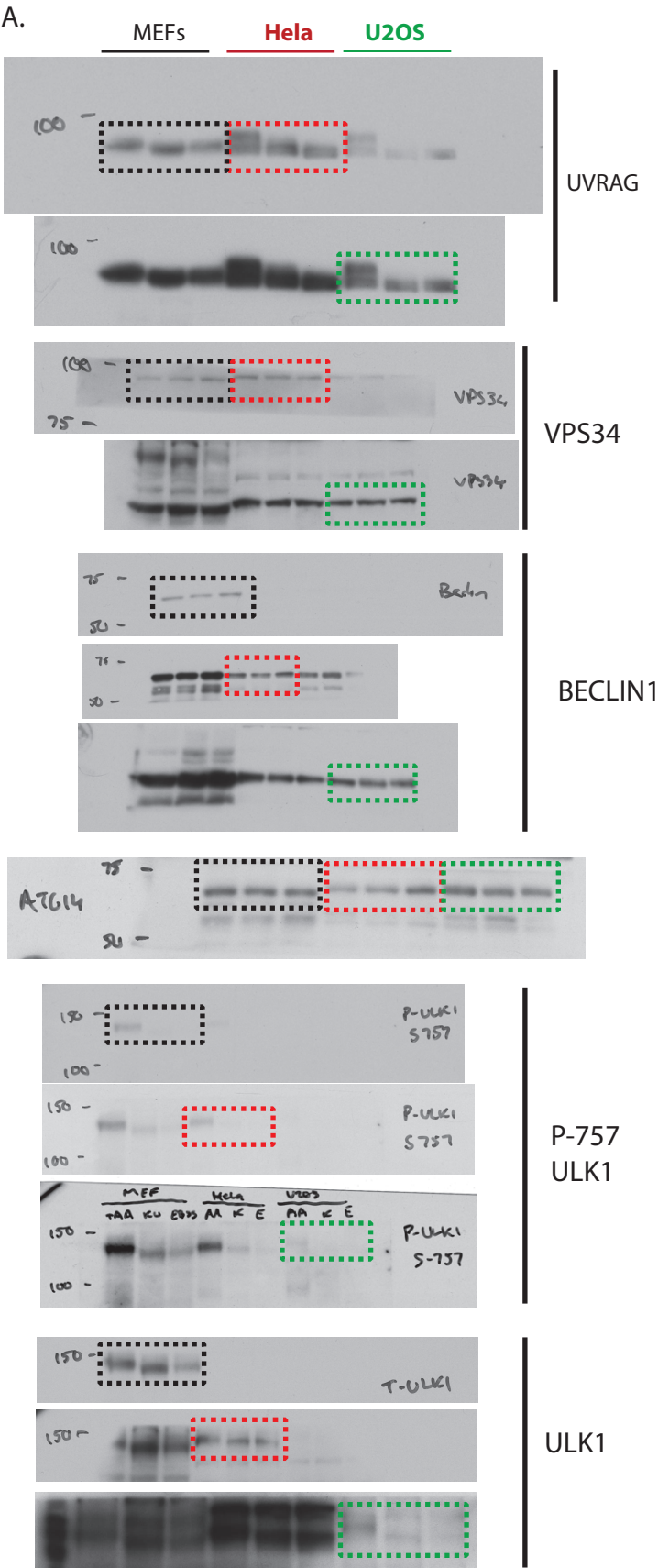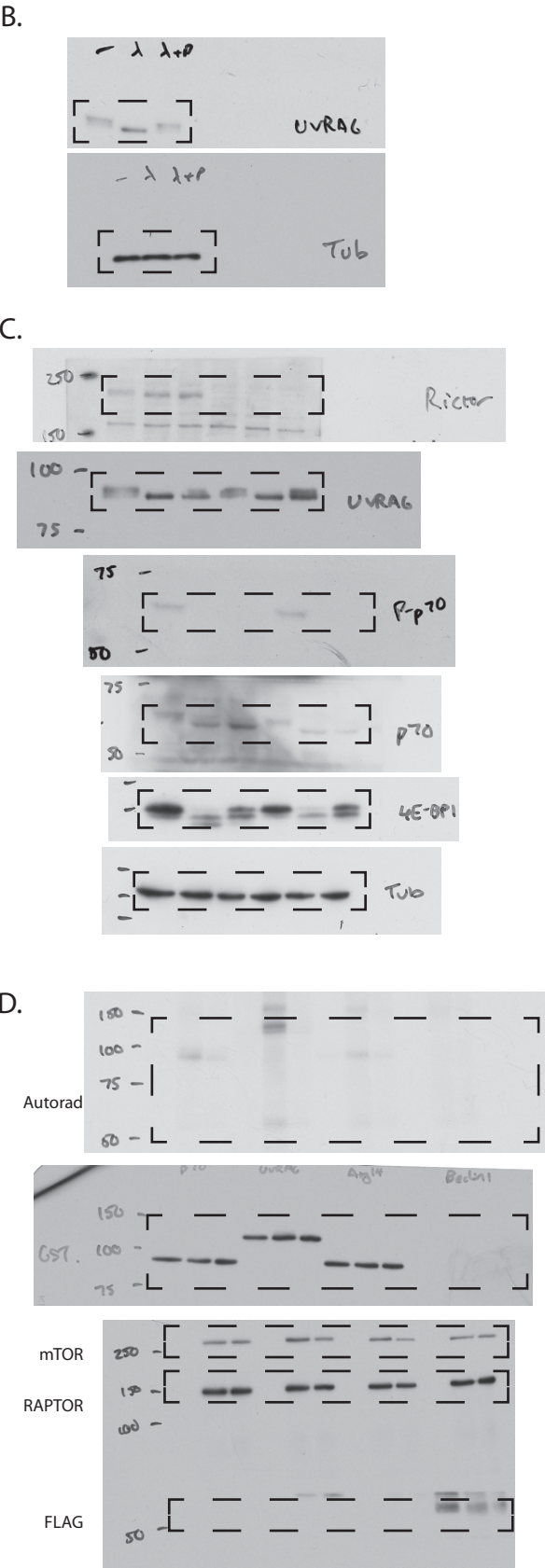

Supplement: Supplementary file 14 [file embj0034-2272-sd14.pdf]

Figure 4 - Source Data

C.

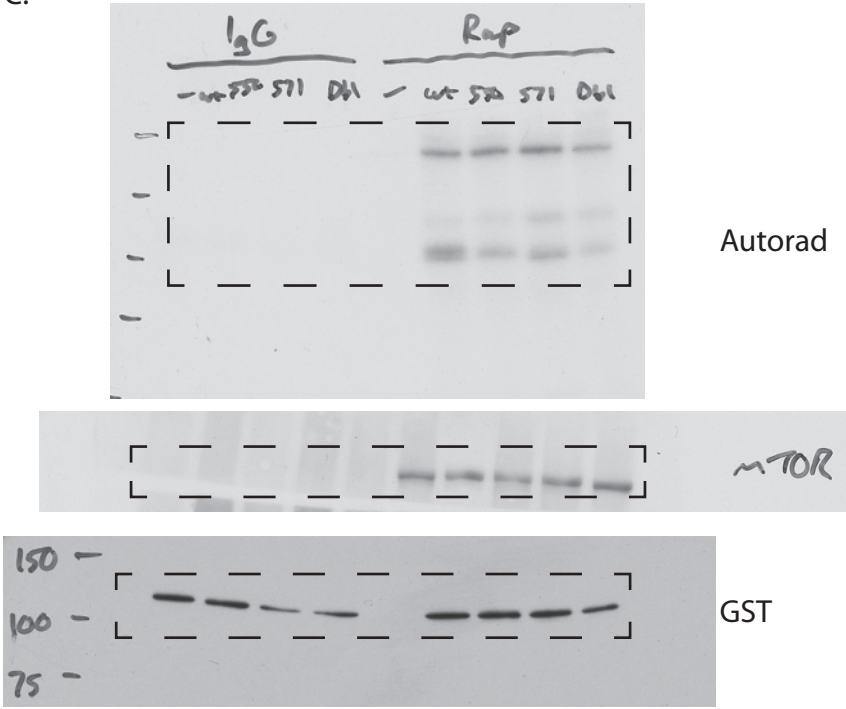

E.

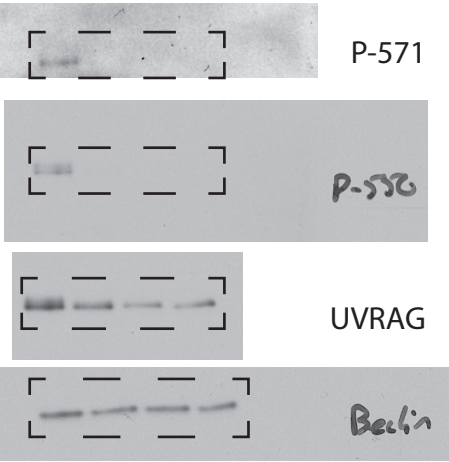

F.

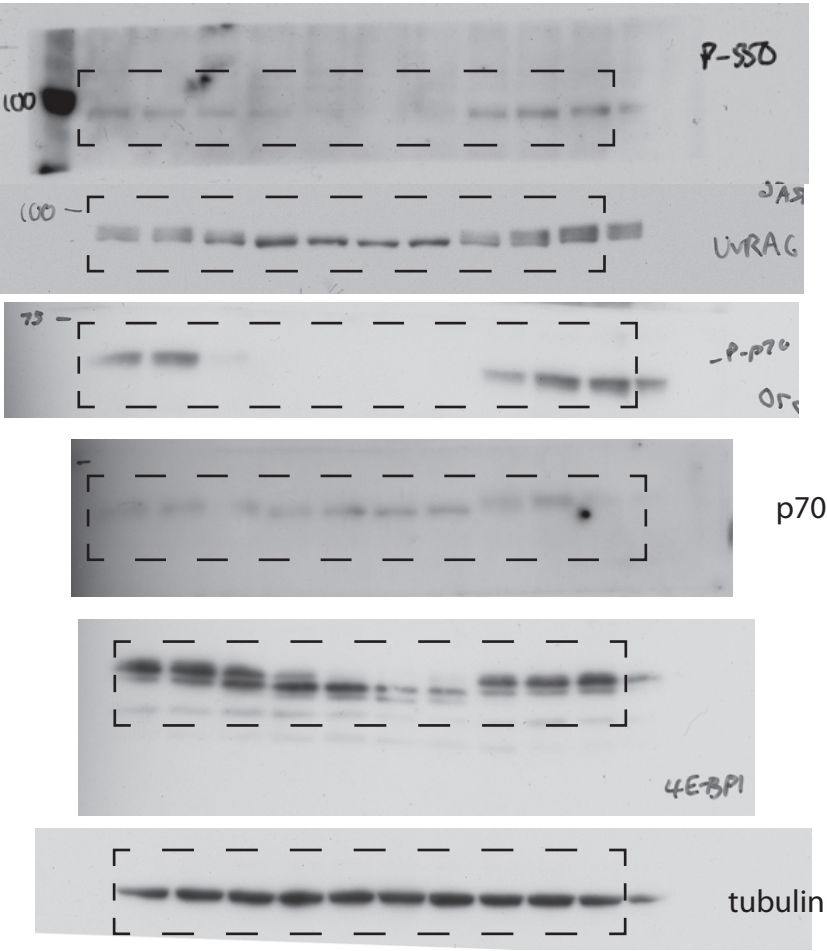

Supplement: Supplementary file 15 [file embj0034-2272-sd15.pdf]

Figure 5 - Source Data

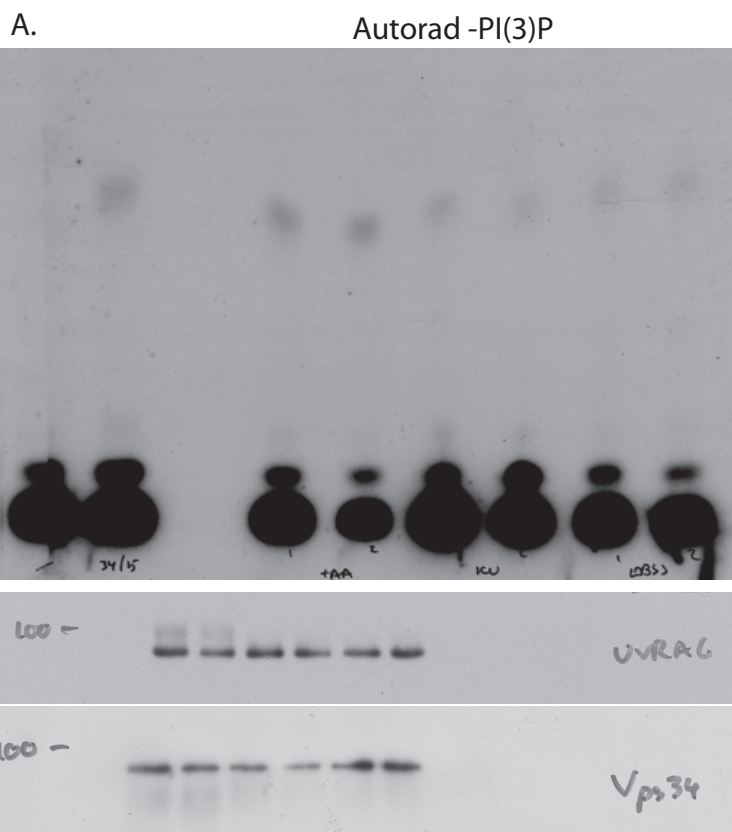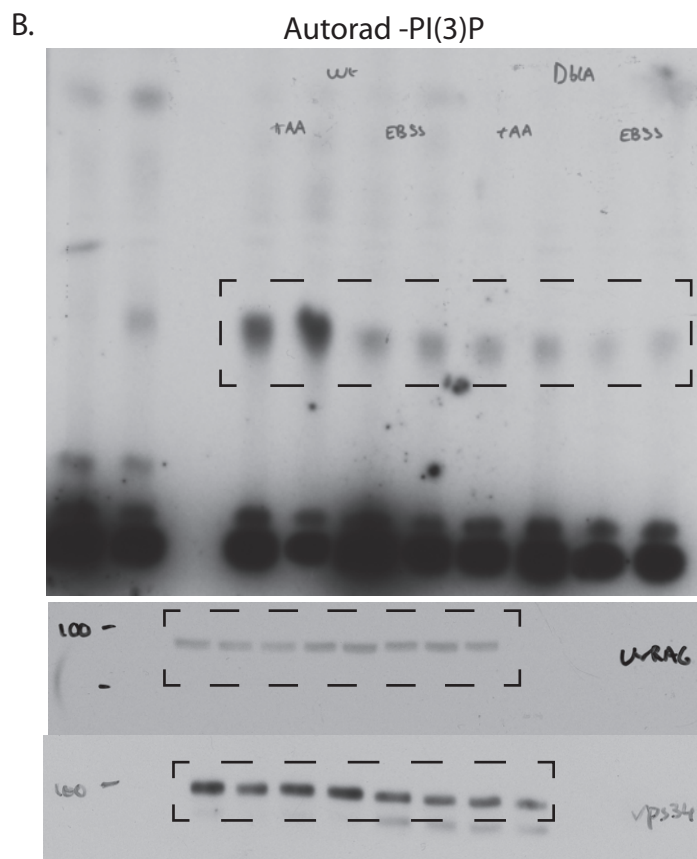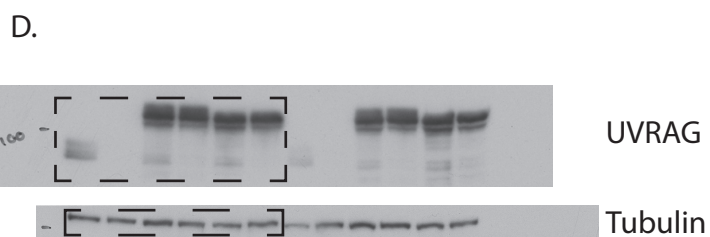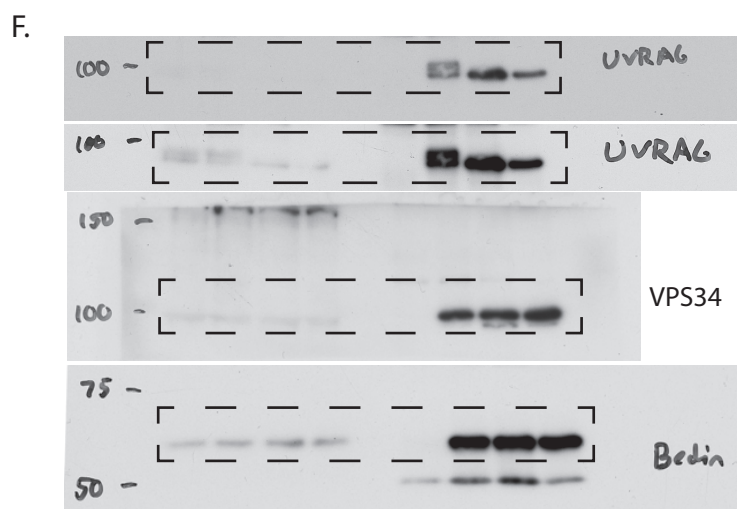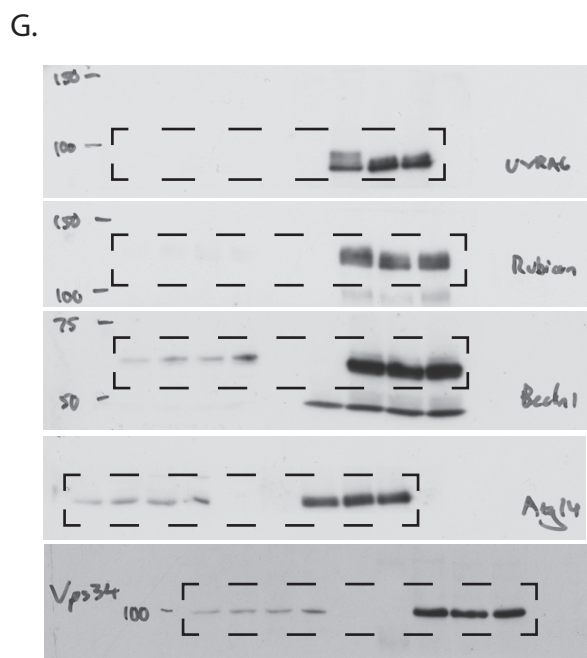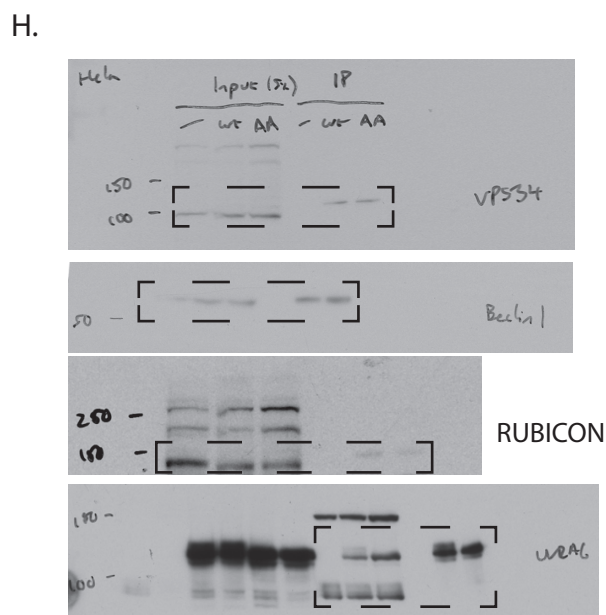

Supplement: Supplementary file 16 [file embj0034-2272-sd16.pdf]

Figure 6 - Source Data

B.

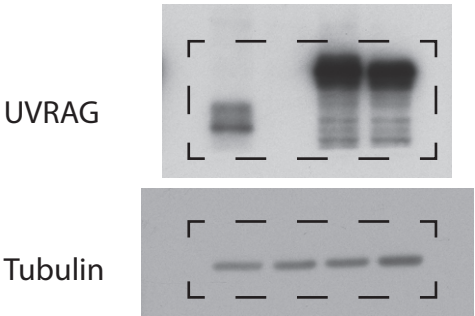

Supplement: Supplementary file 17 [file embj0034-2272-sd17.pdf]

Figure 7 - Source Data

D.

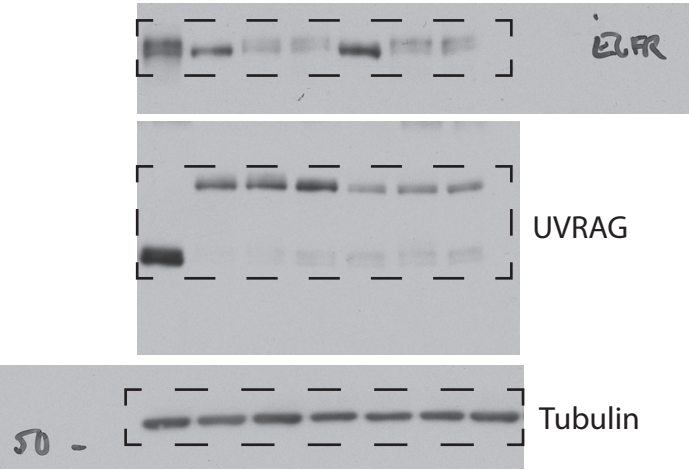

E.

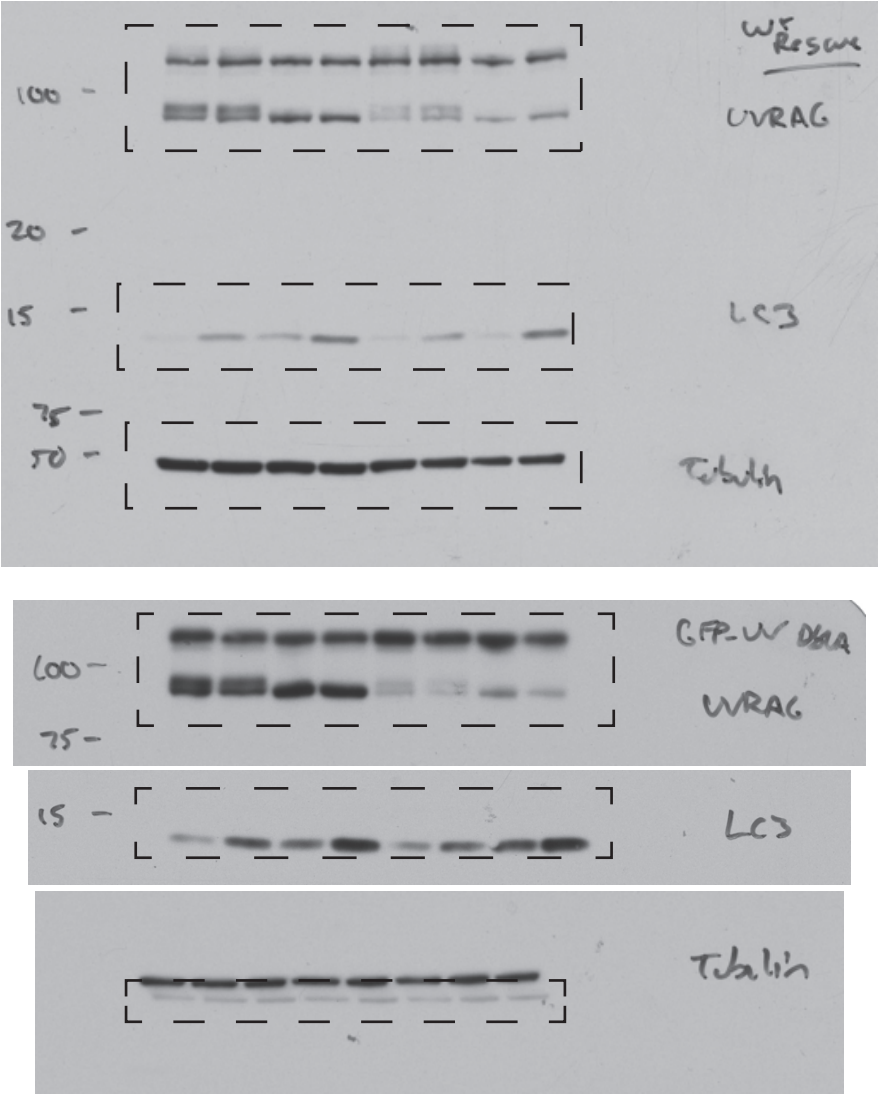

Supplement: Supplementary file 18 [file embj0034-2272-sd18.pdf]

Figure 8 - Source Data

A.

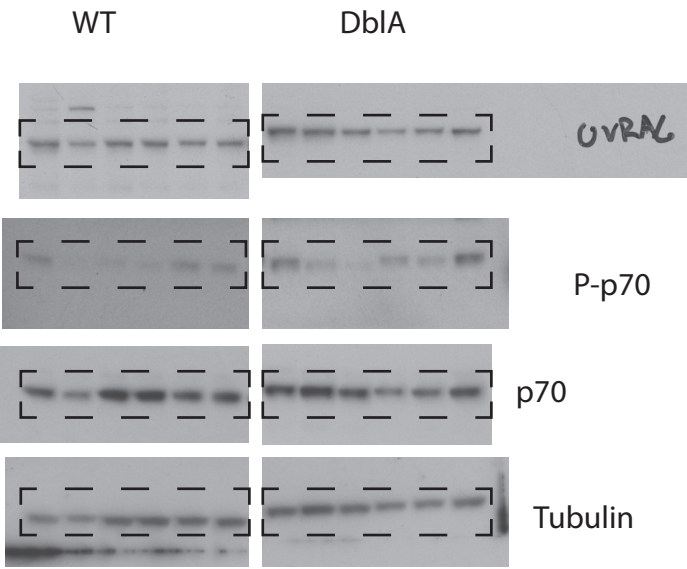

Supplement: Supplementary file 19 [file embj0034-2272-sd19.pdf]
