# Supplementary material for: mTOR activates the VPS34–UVRAG complex to regulate autolysosomal tubulation and cell survival
Source: EMBO J. 2015 Jul 2;34(17):2272–90. doi: 10.15252/embj.201590992 (PMC4585463; doi:10.15252/embj.201590992)
Supplement: Supplementary file 7 [file embj0034-2272-sd7.zip › Movie S1/Supplementary Movie S1 Legend.docx]

**Supplementary Movie S1 – Inhibition of VPS34 causes persistent lysosomal tubulation.**

**(A-B)** U2OS cells stably expressing LAMP1-mCherry were grown in complete media and treated with either **(A)**DMSO or **(B)**1 μM VPS34-IN1 for 1h prior to imaging. Scale bar, 10 μm.
