# Supplementary material for: mTOR activates the VPS34–UVRAG complex to regulate autolysosomal tubulation and cell survival
Source: EMBO J. 2015 Jul 2;34(17):2272–90. doi: 10.15252/embj.201590992 (PMC4585463; doi:10.15252/embj.201590992)
Supplement: Supplementary file 8 [file embj0034-2272-sd8.zip › Movie S2/Supplementary Movie S2 Legend.docx]

**Supplementary Movie S2 – UVRAG phosphorylation regulates lysosomal tubulation.**

**(A-C)** U2OS cells stably expressing LAMP1­‑mCherry (Control - **(A)**), LAMP1-mCherry and GFP-UVRAG wild‑type (WT - **(B)**), or LAMP1-mCherry and GFP-UVRAG S550A+S571A (dblA - **(C)**). Cells in B and C were transfected with UVRAG siRNA 40 h prior to live cell imaging in complete media. Scale bar, 10 μm.
