# Supplementary material for: mTOR activates the VPS34–UVRAG complex to regulate autolysosomal tubulation and cell survival
Source: EMBO J. 2015 Jul 2;34(17):2272–90. doi: 10.15252/embj.201590992 (PMC4585463; doi:10.15252/embj.201590992)
Supplement: Supplementary file 9 [file embj0034-2272-sd9.docx]

**SUPPLEMENTARY FIGURE LEGENDS**

**Figure S1 – PX domain co-localises with EEA1 and CD63.**

**(A-B)** U2OS cells were grown in complete media in the presence or absence of 1 μM VPS34-IN1 prior to fixation and staining for PI(3)P utilising the PX domain conjugate and **(A)** EEA1 or **(B)** CD63. **(C)** Quantitation of PX domain co‑localisation as in (A-B) ± SEM for n = 3 independent experiments.

**Figure S2 – LAMP1 tubules co-localise with LAMTOR**

Live cell images of U2OS cells expressing LAMP1-mCherry and GFP-LAMTOR grown in complete media and treated with DMSO or 1 μM VPS34-IN1 as indicated.

**Figure S3 – UVRAG S498 and T518 phosphorylation are not regulated by mTOR.**

**(A**) Extracted ion chromatogram of UVRAG peptide K479-K505 (S498) from MEFs incubated in complete media in the presence or absence of 1 μM KU0063794 (KU). **(B)** *In vitro* kinase assay with endogenous mTORC1 by immunoprecipitation of RAPTOR from HEK293 cells and incubation with GST‑UVRAG wild-type (WT), S498A or T518A. **(C)** Quantitation of (B), mean UVRAG phosphorylation ± SD for n = 2 independent experiments.

**Figure S4 – UVRAG is localised to lysosomes.**

**(A)** U2OS cells or those stably expressing wild-type (WT) or S550A+S571A (dblA) GFP-UVRAG were transfected with 100 nM control or UVRAG siRNA 40 h prior to treatment. Cells were grown in complete media prior to fixation and staining for GM130, EEA1 or CI-MPR. Scale bar, 10 μm. **(B)** U2OS cells expressing wild-type (WT) or S550A+S571A (dblA) GFP-UVRAG and LAMP1-mCherry were grown in complete media and analysed by live cell imaging. Quantitation represents GFP-UVRAG co-localisation with LAMP1-mCherry ±SEM from n = 3 independent experiments.

**Figure S5 – mTOR inhibition does not alter endocytosis.**

**(A)** HeLa and U2OS cells were serum starved in DMEM and lysed at indicated time-points. **(B)** MEF cells were serum starved in DMEM for 2 h prior to addition of complete media and 50 ng/ml EGF in the presence or absence of 1 μM KU0063794 (KU) for indicated time periods. **(C)** Quantitation of (B), mean EGFR protein level relative to time 0 ± SEM for n = 3 independent experiments. **(D)** U2OS cells were incubated in DMEM + 5 μg/ml Transferrin-594 and the presence or absence of 1 μM KU for 1 h. Cells were washed twice and incubated in complete media in the presence or absence of 1 μM KU and fixed at time-points indicated. **(E)** Quantitation of (D), mean transferrin-594 level relative to time 0 ± SEM for n = 3 independent experiments. **(F)**  Cell lysates from (D) were immunoblotted as indicated.

**Figure S6 - Mutation of UVRAG does not impair DNA damage response.**

**(A)** HEK293 cells were transfected with GST-Ku70 with or without GFP‑UVRAG wild-type (WT) or S550A+S571A (dblA) and grown in complete media. Cells were lysed and GST-Ku70 immunoprecipitated with GST-Sepharose beads and blotted as indicated. **(B)** U2OS cells or those stably expressing wild-type (WT) or S550A+S571A (dblA) GFP-UVRAG were transfected with 100 nM control or UVRAG siRNA 40 h prior to treatment. Cells were grown in complete media or serum and glutamine starvation media for 16 h prior to fixation and staining for γ-H2AX. Quantitation represents mean nuclear γ-H2AX fluorescence per cell ± SEM for n = 3 independent experiments. **(C)** U2OS cells expressing wild-type (WT) or S550A+S571A (dblA) GFP-UVRAG were exposed to laser micro irradiation followed by fixation and immunofluorescent staining (1h after damage) for GFP and γ-H2AX.
