# Supplementary material for: mTOR activates the VPS34–UVRAG complex to regulate autolysosomal tubulation and cell survival
Source: EMBO J. 2015 Jul 2;34(17):2272–90. doi: 10.15252/embj.201590992 (PMC4585463; doi:10.15252/embj.201590992)
Supplement: Supplementary file 13 [file embj0034-2272-sd13.pdf]

THE
EMBO
JOURNAL

Manuscript EMBO-2015-90992

mTOR activates the VPS34-UVRAG complex to regulate autolysosomal tubulation and cell survival

Michael J Munson, George FG Allen, Rachel Toth, David G Campbell, John M Lucocq and Ian G Ganley

Corresponding author: Ian Ganley, University of Dundee

Review timeline:

Submission date:	11 January 2015
Editorial Decision:	02 February 2015
Revision received:	16 May 2015
Accepted:	04 June 2015

Editor: Andrea Leibfried

Transaction Report:

(Note: With the exception of the correction of typographical or spelling errors that could be a source of ambiguity, letters and reports are not edited. The original formatting of letters and referee reports may not be reflected in this compilation.)

1st Editorial Decision 02 February 2015

Thank you for submitting your manuscript entitled 'mTOR activates the VPS34-UVRAG complex to regulate lysosomal tubulation and cell survival'. I have now received reports from all referees, which are enclosed below.

As you will see, the referees find your study interesting. However, they raise a number of concerns, which need to be addressed to better support your conclusions. I will not list all concerns here, but the referees point out that some of the data are too preliminary at this stage and need further validation and support. Furthermore, it is not clear, whether the described signaling pathway specifically acts on lysosomes or on autolysosomes, and it would thus be important to show whether the described regulation occurs during autophagic lysosomal reformation.
Given the comments provided, I would like to invite you to submit a revised version of the manuscript. Please contact me in case of other questions regarding the revision of your manuscript.

When preparing your letter of response to the referees' comments, please bear in mind that this will form part of the Review Process File, and will therefore be available online to the community. For more details on our Transparent Editorial Process, please visit our website: http://emboj.embopress.org/about#Transparent_Process

We generally allow three months as standard revision time. As a matter of policy, competing manuscripts published during this period will not negatively impact on our assessment of the conceptual advance presented by your study. However, we request that you contact me as soon as possible upon publication of any related work, to discuss how to proceed. Should you foresee a problem in meeting this three-month deadline, please let us know in advance and we may be able to grant an extension.

© European Molecular Biology Organization

Thank you for the opportunity to consider your work for publication. I look forward to your revision.

REFEREE REPORTS

Referee #1:

Munson et al. report that class III PI3K produces a lysosomal pool of PI(3)P that counteracts mTOR-dependent lysosomal tubulation. The authors identify two mTOR phosphorylation sites in the class III PI3K subunit UVRAG and provide evidence that UVRAG phosphorylation activates the catalytic activity of the PI3K complex. Mutation of these phosphorylation sites was found to inhibit PI3K lipid kinase activity and resulted in increased lysosomal tubulation. Under long-term starvation conditions, expression of a phosphorylation defective UVRAG mutant (under conditions when endogenous UVRAG was depleted) was found to induce apoptotic cell death. The authors conclude that tubulation dependent autophagosome-lysosome-reformation (ALR) is important for cell survival under nutrient stress and that such tubulation is regulated by class III PI3K. The reported ability of mTOR to regulate class III PI3K via UVRAG phosphorylation is novel and interesting, and so is the reported ability of this PI3K to regulate lysosomal tubulation and cell survival. However, whilst the phosphorylation of UVRAG by mTOR is convincingly demonstrated, some of the cell biological data are more preliminary.

Major points:

1. A central point in this manuscript is the existence of a pool of PI(3)P-containing lysosomes. However, this pool is poorly characterized. All we learn from Fig 1 is that a few structures co-stain for a PI(3)P probe and LAMP1, but the identity of these structures remains elusive. LAMP1 is hardly a specific marker of lysosomes, it is also abundant on late endosomes and can to small extent be found even on early endosomes. The authors therefore need to characterize the PI(3)P-containing LAMP1 structures much more closely, preferably by electron microscopy and the inclusion of additional markers.

2. Related to the previous point, ALR involves tubulation of autolysosomes, not lysosomes as such. In order to test their hypothesis, the authors should investigate directly whether UVRAG controls autolysosome tubulation and ALR.

Minor point:

In Fig. 4B it appears that there is less VPS34 co-immunoprecipitated with dblA than with WT UVRAG. Could this explain the decrease in PI(3)P formation?

Referee #2:

Munson and colleagues have discovered two phosphorylation sites in UVRAG which they show are sites of mTOR phosphorylation and which they propose to regulate Vps34 complex (containing UVRAG and not ATG14) kinase activity. The link which led to this discovery was somewhat indirect as they observed tubulation of lysosomes at low concentrations of the recently described "VPS34IN" which required mTOR activity. They further propose that a pool of PI3P produced by activation of UVRAG by mTOR is produced on lysosomes which regulates tubulation. Finally they provide data which suggest disruption of tubulation which occurs as part of ALR causes cell death. Overall the data are robust and technically sound and support the authors conclusions. While this is not a criticism of the manuscript, in my opinion the number of discrepancies in the literature and lack of clarity about the function of UVRAG, and the potential complexity of the phosphoinositides on the lysosome make any absolute conclusions about the role of these phosphorylations sites difficult. The authors have done a good job in defining a phenotype, but I am not 100% convinced the data is sufficient to propose the model in Fig. 8. and in addition, as detailed below, more thorough examination of the cause of cell death needs to be done.

Points;
1. Fig. 2C, it appears as if there is a small amount of Beclin1 phosphorylated in UVRAG immunoprecipitation, and the text should be corrected. GST-UVRAG will bind robust amounts of endogenous Beclin1.
2. Fig. 4A and B. please show immunoprecipitated endogenous UVRAG and FLAG-UVRAG. Fig. 4F-G what cell type was used?
3. Fig. 5A please show depletion and expression of GFP-WT and dblA UVRAG using anti-UVRAG antibody.
4. Fig. 5B. It is not clear what alteration of lysosome morphology is oberved or if there is disruption to LAMP1 trafficking. The LAMP-positive puncta size varies widely between conditions. In addition, it appears if LAMP1 is on the plasma membrane in the dblA rescue. How was the puncta counting done and in particular the threshold for size and intensity? it looks as if there are fewer normal LAMP1 puncta in the dblA and a mislocalization. Besides lysotracker are the LAMP1 structures positive for any other lysosomal markers? CD63, V-ATPASE?
5. Zhao et al Dev Cell 2012 showed that UVRAG interacts with DNA-PK and is required for chromosome stability. The region of UVRAG that interacts with DNA-PK is where the mTOR phosphorylation cites are located the authors should test if cell death occurs due to the disruption of this interaction in the doubleA mutants. The starvation conditions could simply place the cells under extreme stress. These experiments should be done more carefully looking at chromosome stability, cell division and detail how the cells are dying.

Referee #3:

In this manuscript, the authors described the role of mTOR mediated UVRAG phosphorylation in lysosome tubulation, and the physiological role of mTOR mediated UVRAG phosphorylation in cells undergoing long time starvation.
Overall speaking, this study looks interesting and for most part the data are compelling.

Specific comment:

1) Tubular lysosomes have been described previously, but at this moment, whether the molecular regulation of lysosome tubulation and ALR (which is autolysosome tubulation) are identical still remains to be determined. It is very clear that mTOR mediated UVRAG phosphorylation regulates lysosome tubulation, however, it is less clear to me whether it affects autophagic lysosome reformation (ALR). The authors should test how the UVRAG mutant affects ALR in the experimental setting described in the original ALR paper (serum/glutamine starvation). The authors may also want to discuss the similarity and difference between these two processes a little bit more.
2) The link between persistent lysosome tubulation and increased cell death in UVRAG mutant cells undergoing long time starvation is relatively weak. The authors either need to modify their statement or provide some mechanistic insight to explain how persistently lysosome tubulation causes cell death. Is lysosome function impaired in UVRAG mutant cells undergoing long time starvation?
3) Figure 1, besides the PX staining, the authors may want to verify the reduction of PI3P using biochemical assays.
4) Is UVRAG present on lysosomes or autolysosomes?
5) Figure 1D needs statistic analysis.
6) Figure 4C, it is clear that the PI3P level is reduced in UVRAG KD cells, however, the authors should also measure the reduction of PI(3)P on the lysosome population using PX/LAMP1 staining.
7) Figure 5A, the EEA staining does look a little bit abnormal in siUVRAG. To avoid confusion, the author should choose more representative images.
8) In Fig.7B, there is no scale bar.

1st Revision - authors' response 16 May 2015

Referee #1:

Munson et al. report that class III PI3K produces a lysosomal pool of PI(3)P that counteracts mTOR-dependent lysosomal tubulation. The authors identify two mTOR phosphorylation sites in the class III PI3K subunit UVRAG and provide evidence that UVRAG phosphorylation activates the

catalytic activity of the PI3K complex. Mutation of these phosphorylation sites was found to inhibit PI3K lipid kinase activity and resulted in increased lysosomal tubulation. Under long-term starvation conditions, expression of a phosphorylation defective UVRAG mutant (under conditions when endogenous UVRAG was depleted) was found to induce apoptotic cell death. The authors conclude that tubulation dependent autophagosome-lysosome-reformation (ALR) is important for cell survival under nutrient stress and that such tubulation is regulated by class III PI3K. The reported ability of mTOR to regulate class III PI3K via UVRAG phosphorylation is novel and interesting, and so is the reported ability of this
PI3K to regulate lysosomal tubulation and cell survival. However, whilst the phosphorylation of UVRAG by mTOR is convincingly demonstrated, some of the cell biological data are more preliminary.

We thank the reviewer for their support and comments to improve the manuscript.

Major points:

1. A central point in this manuscript is the existence of a pool of PI(3)P-containing lysosomes. However, this pool is poorly characterized. All we learn from Fig 1 is that a few structures co-stain for a PI(3)P probe and LAMP1, but the identity of these structures remains elusive. LAMP1 is hardly a specific marker of lysosomes, it is also abundant on late endosomes and can to small extent be found even on early endosomes. The authors therefore need to characterize the PI(3)P-containing LAMP1 structures much more closely, preferably by electron microscopy and the inclusion of additional markers.

We thank the reviewer for the suggestions and agree that LAMP1 does not necessarily mean lysosomal localization. To address this we have used the additional lysosomal markers LAMTOR and CD63 (shown in revised Figure 1 and E1), which show a very similar staining pattern to LAMP1 and strengthen the argument for lysosomal localization. We have also carried out immuno-EM using the recombinant GST-PX and secondary gold anti-GST and found profiles that resemble lysosomes which label for PI(3)P. Due to antibody technicalities we were not able to perform a double labeling experiment, but found in parallel that LAMP1 also labeled similar lysosomal-like structures. This new data is shown in Figure 1D. We hope, together with the additional endogenous markers of lysosomes shown by light microscopy, this strengthens our argument that PI(3)P is present on lysosomes, even if it is only a small population at any one time.

2. Related to the previous point, ALR involves tubulation of autolysosomes, not lysosomes as such. In order to test their hypothesis, the authors should investigate directly whether UVRAG controls autolysosome tubulation and ALR.

Thank you for this suggestion as it is an important point. To look specifically at autolysosomes, we stably transduced cells expressing LAMP1-GFP with mCherry-LC3, with the rational that basal autophagy should lead to the accumulation of the relatively stable mCherry in lysosomes. In support of this we found that a large proportion of mCherry-LC3 co-localised with LAMP1-GFP, and when we inhibited VPS34, almost all (90%) of the tubules emanated from these double labeled structures, or contained both markers. We feel this is strong evidence that it is autolysosomes that are tubulating. The new data are shown in revised Figure 2C.

Minor point:

In Fig. 4B it appears that there is less VPS34 co-immunoprecipitated with dblA than with WT UVRAG. Could this explain the decrease in PI(3)P formation?

We do not think this is the case and apologize for the confusion with the example blots that were shown. In this example shown there was indeed slightly less VPS34 co-IP'd, but all activities were normalized to the amount of VPS34 in the assay. The experiment was carried out with four biological replicates to get accurate quantitation and we have replaced the blots with another experiment to avoid confusion. We do not think less VPS34 associates with the dblA mutant compared to the WT (see endogenous and exogenous IPs shown in Figure 5F-H).

© European Molecular Biology Organization

Referee #2:

Munson and colleagues have discovered two phosphorylation sites in UVRAG which they show are sites of mTOR phosphorylation and which they propose to regulate Vps34 complex (containing UVRAG and not ATG14) kinase activity. The link which led to this discovery was somewhat indirect as they observed tubulation of lysosomes at low concentrations of the recently described "VPS34IN" which required mTOR activity. They further propose that a pool of PI3P produced by activation of UVRAG by mTOR is produced on lysosomes which regulates tubulation. Finally they provide data which suggest disruption of tubulation which occurs as part of ALR causes cell death. Overall the data are robust and technically sound and support the authors conclusions. While this is not a criticism of the manuscript, in my opinion the number of discrepancies in the literature and lack of clarity about the function of UVRAG, and the potential complexity of the phosphoinositides on the lysosome make any absolute conclusions about the role of these phosphorylations sites difficult. The authors have done a good job in defining a phenotype, but I am not 100% convinced the data is sufficient to propose the model in Fig. 8. and in addition, as detailed below, more thorough examination of the cause of cell death needs to be done.

We thank the reviewer for their careful analysis of our manuscript and agree the situation in the current literature is complicated. At the least, the multiple roles for UVRAG highlight its importance in acting as a node to control multiple cellular processes. As mentioned in the discussion the multiple phosphoinositides could provide distinct timing mechanisms to control the different stages of tubulation. We also now reference work from the Gallop and Kirschner groups showing co-operation between PI(3)P and PI(4,5)P_2 to drive actin polymerization at membranes, so evidence for multiple phosphoinositides in a given process is mounting. We are far from understanding this process, but we feel this manuscript is important in establishing a new and unexpected role for the VPS34-UVRAG complex in ALR and also providing mechanistic insight into how mTOR regulates it. The data fit the model proposed, but we agree they would also fit other unrelated models. We are now working to test our model further in a follow-up study.

Points;

1. Fig. 2C, it appears as if there is a small amount of Beclin1 phosphorylated in UVRAG immunoprecipitation, and the text should be corrected. GST-UVRAG will bind robust amounts of endogenous Beclin1.

We thank the reviewer for this observation and have now added the statement below in describing what is now Figure 3D:
"Interestingly, we did observe a phosphorylated band at the position of BECLIN1 in the UVRAG samples, which could imply that mTOR can phosphorylate Beclin1 when it is part of the UVRAG complex. Further work will be needed to clarify this."

2. Fig. 4A and B. please show immunoprecipitated endogenous UVRAG and FLAG-UVRAG. Fig. 4F-G what cell type was used?

Data has now been included in what is now revised Figure 5A and B. The cell type used was U2OS and this description has now been added to the Figure legend.

3. Fig. 5A please show depletion and expression of GFP-WT and dblA UVRAG using anti-UVRAG antibody.

We apologize for the omission and the levels of expression are now shown in revised Figure 6B.

4. Fig. 5B. It is not clear what alteration of lysosome morphology is oberved or if there is disruption to LAMP1 trafficking. The LAMP-positive puncta size varies widely between conditions. In addition, it appears if LAMP1 is on the plasma membrane in the dblA rescue. How was the puncta counting done and in particular the threshold for size and intensity? it looks as if there are fewer normal LAMP1 puncta in the dblA and a mislocalization. Besides lysotracker are the LAMP1 structures positive for any other lysosomal markers? CD63, V-ATPASE?

We have now added more quantitation in terms of number and size of LAMP1 structures. The details on how this was carried out have been added to the methods section and shown here:

"Immunofluorescence images were quantified for cell intensity, punctate structures and co-localisation utilising NIS Elements. Automated counting was carried out by intensity thresholding, the criteria set was kept consistent within experiments between conditions and checked thoroughly to ensure accurate representation of the data."
Upon averaging the data out there appears to be little statistical significance between the sizes of LAMP1 structures under the different conditions, only the number of structures. Given the disruption of tubules upon fixation, we do not want to draw too many conclusions from this data alone in terms of mislocalisation. From the live cell microscopy (see Figure 7A) the structures appear to be dispersed throughout the cytosol and it is possible that there is an increase in plasma membrane staining – but if it is, it is in very small, localized patches that resemble vesicles, which label for Lysotracker. We have not ruled out that some LAMP1 is mislocalised to other compartments, but the staining pattern does look distinct from Golgi, early and late endosomes (as is now shown in revised Figure E6).
We appreciate the comments about additional lysosomal markers for the LAMP1 structures and now show, in addition to Lysotracker, that GFP-LAMTOR and mCherry-LC3 also form tubules, implying that they are autolysosomal in nature (see data in revised Figure 2C and E2). Additionally, we have looked at more endogenous markers in fixed cells including CD63 and LAMTOR as well as the original LAMP1 and show that they co-localise with the recombinant PX domain (see revised Figure 1B and C). We also now include immuno-EM to show that lysosomal structures contain PI(3)P (using the PX domain – see revised Figure 1D and E).

5. Zhao et al Dev Cell 2012 showed that UVRAG interacts with DNA-PK and is required for chromosome stability. The region of UVRAG that interacts with DNA-PK is where the mTOR phosphorylation cites are located the authors should test if cell death occurs due to the disruption of this interaction in the doubleA mutants. The starvation conditions could simply place the cells under extreme stress. These experiments should be done more carefully looking at chromosome stability, cell division and detail how the cells are dying.

We thank the reviewer for this suggestion as an alternate mode of increased cell death. To address this we looked to see if WT or dblA UVRAG showed any difference in the reported binding to Ku70 and DNA-PK indictated in the Zhao et al paper. We did find a very weak interaction of Ku70 with UVRAG, but this was the same for WT of the dblA mutant, thus we concluded that the interaction could explain the difference in cell death observed with the dblA mutant (see Figure E8). Next we looked more directly at DNA damage and found no significant increase in g-H2AX staining under the starvation conditions used in the different UVRAG-expressing cells. Likewise, we carried out "laser stripe" induced DNA damage assays as in the Zhao et al paper, but were unable to detect recruitment of either WT or dblA UVRAG to sites of damage (as indicated by the g-H2AX staining). We do not dispute the findings of Zhao et al, but conclude, as we don't see then same observations our system, this mode of UVRAG function is unlikely to explain our results. These data are shown in the new Figure E8.
We still do not know the mechanism of cell death but have added more data to support our current hypothesis of lysosome disruption. mTOR inhibition, which prevents tubule formation, rescues the dblA mutant cell death phenotype (see new data in revised Figure 8D and E). Additionally, we have found that the dblA mutants are more sensitive to lysosomal damage using LLoME (Figure 8F and G). Taken together, we believe this provides a good basis for further follow-up experiments to confirm that lysosomal tubulation and function is impaired under these longer-term starvation conditions; which we hope to carry out in follow-up studies.

Referee #3:

In this manuscript, the authors described the role of mTOR mediated UVRAG phosphorylation in lysosome tubulation, and the physiological role of mTOR mediated UVRAG phosphorylation in cells undergoing long time starvation.
Overall speaking, this study looks interesting and for most part the data are compelling.

We thank the reviewer for their comments.

Specific comment:

© European Molecular Biology Organization

1) Tubular lysosomes have been described previously, but at this moment, whether the molecular regulation of lysosome tubulation and ALR (which is autolysosome tubulation) are identical still remains to be determined. It is very clear that mTOR mediated UVRAG phosphorylation regulates lysosome tubulation, however, it is less clear to me whether it affects autophagic lysosome reformation (ALR). The authors should test how the UVRAG mutant affects ALR in the experimental setting described in the original ALR paper (serum/glutamine starvation). The authors may also want to discuss the similarity and difference between these two processes a little bit more.

We apologize for the confusion, but we did carry out the survival experiments using the serum/glutamine starvation used in the original papers. We have the following statement in the results section" To look at mTOR reactivation we used serum and glutamine starvation in cells depleted of endogenous UVRAG and expressing either siRNA resistant WT or dblA GFP-UVRAG".
To clarify whether this tubulation is acting at the level of ALR, we stably expressed mCherry-LC3 and looked at the co-localisation with LAMP1-GFP. This point was also raised by reviewer 1 and as mentioned earlier, we found that upon VPS34 inhibition, almost all (90%) of the tubules emanated from these double labeled structures, or contained both markers. We feel this is strong evidence that it is autolysosomes that are tubulating. The fact that this tubulation happens under "basal" conditions implies that ALR is perhaps constantly occurring, just that under normal conditions it is not as evident. We have added to the discussion a little bit more about the similarities and differences as requested:

"The autolysosomal tubulation observed here differs somewhat for the original observations of ALR (Yu et al., 2010). Firstly, we find tubulation occurs under normal nutrient rich growth conditions. We take this to mean that ALR is constantly occurring, but under these basal conditions it is not as evident as those following longer-term starvation. The fact that we are blocking this process by VPS34 inhibition allows us to more easily visualise it here. Secondly, the classical ALR tubules are not positive for Lysotracker or cargo such as LC3, where as the tubules described here are positive for both. A potential explanation for this may partially due to the persistent nature of the tubules: their prolonged existence may mean it is more likely that the quality control mechanisms to prevent passage of cargo into tubules is overcome. Further work will be needed to clarify this situation."

2) The link between persistent lysosome tubulation and increased cell death in UVRAG mutant cells undergoing long time starvation is relatively weak. The authors either need to modify their statement or provide some mechanistic insight to explain how persistently lysosome tubulation causes cell death. Is lysosome function impaired in UVRAG mutant cells undergoing long time starvation?

We thank the reviewer for this suggestion to strengthen our conclusions. Reviewer 2 also raised similar concerns and suggested DNA damage as a potential mechanism, given a recently published paper by Zhao et al., As mentioned above we looked at this and the data suggest this is not the case. To strengthen our argument that lysosomal tubulation may be involved we inhibited mTOR, which prevents tubule formation, and found this rescues the dblA mutant cell death phenotype (see new data in revised Figure 8D and E). Additionally, we have found that the dblA mutants are more sensitive to lysosomal damage using LLoME (Figure 8F and G). We do admit that this is still correlative and not definitive proof, but we feel the supporting evidence is strong and provides us with a good foundation to warrant follow-up studies to clarify this.
We also thank the reviewer for the suggestion to look at lysosomal function under these longer starvation conditions. Unfortunately, because of the large degree of cell death observed in the dblA cells at these time points, this makes it difficult to draw any firm conclusions.

3) Figure 1, besides the PX staining, the authors may want to verify the reduction of PI3P using biochemical assays.

We agree with the reviewer that it would be advantageous to use a different method to quantify cellular PI(3)P. However, my understanding is that it is technically very challenging to carry out HPLC or mass spec lipidomics, primarily due to the different fatty acid acyl chain species that can occur in these phosphoinositides. We certainly do not have the expertise or equipment to carry out these experiments in house and I think this would require a lengthy collaboration. We do feel that the PX domain staining assay, combined with the in vitro lipid kinase assays does provide convincing evidence that PI(3)P levels are altered by mTOR inhibition of UVRAG phosphorylation.

4) Is UVRAG present on lysosomes or autolysosomes?

We thank the reviewer for this suggestion and found that approximately 50% of GFP-UVRAG did indeed co-localise with LAMP1-mCherry (these data are now shown in Figure E6B). We feel this further strengthens the argument that VPS34-UVRAG is acting at the lysosome.

5) Figure 1D needs statistic analysis.

We apologize for the omission. Quantitation of the tubule number and length is now shown in the revised Figure 2B. As can be seen the data show a significant increase in size and number of tubules, which are blocked by mTOR inhibition.

6) Figure 4C, it is clear that the PI3P level is reduced in UVRAG KD cells, however, the authors should also measure the reduction of PI(3)P on the lysosome population using PX/LAMP1 staining.

This is a good suggestion and we carried out the experiment and show the data in revised Figure 6D and E. These support our conclusions and clearly show that there is significantly less PX domain co-localising with LAMP1 in the dblA UVRAG mutant cells.

7) Figure 5A, the EEA staining does look a little bit abnormal in siUVRAG. To avoid confusion, the author should choose more representative images.

We apologize for this and now include new panels for EEA1 in what is now Figure E6. As we originally claimed, the staining pattern of EEA1 is similar across all the conditions.

8) In Fig.7B, there is no scale bar.

This has now been included on what is the revised Figure 8.

Accepted 04 June 2015

Thank you for submitting your revised manuscript for our consideration. It has now been seen once more by the original referees (see comments below), who are all satisfied with the revisions and therefore have no further objections towards publication in The EMBO Journal. I am thus happy to accept your manuscript for publication.

Thank you very much for your contribution to our journal!

REFEREE REPORTS

Referee #1:

The authors have successfully addressed the points I raised, and the revised manuscript is much improved. I am happy to recommend publication in the EMBO Journal.

Referee #2:

The authors have addressed all my comments.

Referee #3:

Authors have addressed my comments satisfactorily. Congratulations for this beautiful work.

© European Molecular Biology Organization
